# *Saccharomyces cerevisiae* Tti2 Regulates PIKK Proteins and Stress Response

**DOI:** 10.1534/g3.116.029520

**Published:** 2016-04-05

**Authors:** Kyle S. Hoffman, Martin L. Duennwald, Jim Karagiannis, Julie Genereaux, Alexander S. McCarton, Christopher J. Brandl

**Affiliations:** * Department of Biochemistry, Schulich School of Medicine and Dentistry, The University of Western Ontario, London, Ontario, N6A5C1 Canada; † Department of Pathology, Schulich School of Medicine and Dentistry, The University of Western Ontario, London, Ontario, N6A5C1, Canada; ‡ Department of Biology, The University of Western Ontario, London, Ontario, N6A5B7 Canada

**Keywords:** TTT complex, PIKK proteins, heat shock response, chaperone, protein expression

## Abstract

The TTT complex is composed of the three essential proteins Tel2, Tti1, and Tti2. The complex is required to maintain steady state levels of phosphatidylinositol 3-kinase-related kinase (PIKK) proteins, including mTOR, ATM/Tel1, ATR/Mec1, and TRRAP/Tra1, all of which serve as regulators of critical cell signaling pathways. Due to their association with heat shock proteins, and with newly synthesized PIKK peptides, components of the TTT complex may act as cochaperones. Here, we analyze the consequences of depleting the cellular level of Tti2 in *Saccharomyces cerevisiae*. We show that yeast expressing low levels of Tti2 are viable under optimal growth conditions, but the cells are sensitive to a number of stress conditions that involve PIKK pathways. In agreement with this, depleting Tti2 levels decreased expression of Tra1, Mec1, and Tor1, affected their localization and inhibited the stress responses in which these molecules are involved. Tti2 expression was not increased during heat shock, implying that it does not play a general role in the heat shock response. However, steady state levels of Hsp42 increase when Tti2 is depleted, and *tti2_L187P_* has a synthetic interaction with exon 1 of the human Huntingtin gene containing a 103 residue polyQ sequence, suggesting a general role in protein quality control. We also find that overexpressing Hsp90 or its cochaperones is synthetic lethal when Tti2 is depleted, an effect possibly due to imbalanced stoichiometry of a complex required for PIKK assembly. These results indicate that Tti2 does not act as a general chaperone, but may have a specialized function in PIKK folding and/or complex assembly.

The biosynthesis and regulation of phosphatidylinositol 3-kinase-related kinase (PIKK) proteins is crucial for cells to grow, proliferate, and respond to stress conditions. The PIKK family of proteins includes ATM/Te11 (mammalian/yeast), ATR/Mec1, mTOR/Tor1, Tor2, TRRAP/Tra1, and the mammalian proteins DNA-dependent protein kinase (DNA-PKcs) and SMG1. Each has important roles in cell signaling during stress (reviewed in Abraham 2004), and regulate one or more critical functions such as cell growth and nutrient response (mTOR), DNA damage response (ATM, ATR, and DNA-PKcs), and the regulation of gene expression (TRRAP and SMG1) (reviewed in Yamashita *et al.* 2005; Cimprich and Cortez 2008; Lovejoy and Cortez 2009; Shimobayashi and Hall 2014). The PIKK proteins are large, and share extensive N-terminal helical regions, and a C-terminal domain that resembles the phosphatidylinositol-3-kinases (Lempiäinen and Halazonetis 2009). Perhaps due to this common structure, biosynthesis and maintenance of the PIKKs are regulated by a common set of proteins. This first became apparent when Takai *et al.* (2007) demonstrated that Tel2 was required to maintain the steady state level of ATM, ATR, DNA-PKcs, SMG1, mTOR, and TRRAP. In subsequent work Takai *et al.* (2010) demonstrated that Tel2 interacts with newly synthesized proteins, suggesting that it executes cotranslational chaperone activity. *TEL2* was also identified in a screen for genes involved in the DNA damage response, likely as a result of its regulation of ATM and ATR (Hurov *et al.* 2010). In the same screen, Hurov *et al.* (2010) identified the genes encoding Tti1 (Tel2 interactor) and Tti2. The three proteins were found to mutually associate and coelute in molecular weight fractions corresponding to a complex they termed the TTT complex. The TTT complex associates with a number of molecular chaperones including Hsp90, Hsp70, Hsp40, and the R2TP/prefoldin-like complex (Hořejší *et al.* 2010; Takai *et al.* 2010). The TTT complex is thus considered a cochaperone, yet the role of each member of the complex, its substrate specificity, and its mechanism of action are unknown.

We identified *tti2* as a genetic suppressor of a *tra1* allele, which alters the C-terminal phenylalanine to alanine (Genereaux *et al.* 2012). This allele reduces the stability and nuclear localization of Tra1, resulting in reduced growth, particularly under stress conditions. Two alleles of *tti2* (Phe328 to Ser and Ile336 to Phe) suppress the *tra1-F3744A* mutation. Consistent with a role for Tti2 as a cochaperone, *tti2* suppression of *tra1-F3744A* reduces its degradation, and enhances the stability and nuclear localization of Tra1-F3744A (Genereaux *et al.* 2012). The *tti2* alleles did not, however, suppress the growth defects of a *mec1* allele with a C-terminal Trp to Ala mutation, suggesting specific interactions between Tti2 and Tra1.

In *Saccharomyces cerevisiae*, *TTI2* encodes an essential protein of 421 amino acid residues. Like Tel2 (Takai *et al.* 2010), Tti2 is predicted to be primarily helical, and localizes to both the nucleus and cytoplasm (Genereaux *et al.* 2012). Further emphasizing the importance of Tti2 are findings that it is implicated in human brain development. A missense mutation that converts I436 to N within human Tti2 causes an autosomal recessive disorder defined by microcephaly, short stature, behavioral problems, skeletal abnormalities, and facial dismorphic features (Langouët *et al.* 2013). Furthermore, A P367L missense mutation in *TTI2* has been linked to intellectual disability (Najmabadi *et al.* 2011). Individuals with a Tti2 defect show characteristics similar to individuals who are unable to respond to DNA damage, possibly due to inhibition of ATR function (Langouët *et al.* 2013).

Our goal is to use a molecular genetic approach to define the role of Tti2, characterize its structure/function relationships, as well as its relationship with Tel2 and Tti1. We therefore began this study with a screen for *tti2* alleles that would identify features required for the function of the protein. Surprisingly, we identified an allele with an ochre mutation at codon 276. As C-terminal truncations of Tti2 do not support viability, this result suggested that low levels of Tti2 potentially obtained by read-through of the ochre mutation were sufficient for viability. To further characterize the effect of depleting Tti2, we placed it under control of the *GAL10* promoter, and analyzed the properties of the protein and strain in raffinose and glucose-containing media. We demonstrate that low levels of Tti2 are sufficient for viability in nonstress conditions. Depleting Tti2 does, however, result in temperature sensitivity, and impairs the ability of cells to respond to certain conditions of stress related to PIKK function. We show that the steady state levels of Mec1, Tra1, and Tor1 decrease when Tti2 levels are depleted, with Mec1 and Tra1 being more affected. A significant proportion of both Tra1 and Mec1 mislocalize to foci within the cytosol when Tti2 is expressed at low levels, which does not appear to result from protein aggregation. Furthermore, overexpressing Hsp90, a molecular chaperone involved in PIKK complex assembly (Hořejší *et al.* 2010; Takai *et al.* 2010), results in synthetic lethality in cells depleted of Tti2. Tti2 is not induced by heat shock, but its absence induces expression of Hsp42. Taken together, these results support a specialized role for Tti2 in PIKK complex assembly, rather than acting as a general chaperone in protein folding.

## Materials and Methods

### Yeast strains and growth

Yeast strains were grown in YP (yeast peptone) media or synthetic dropout media supplemented with required nitrogenous bases and amino acids. Strains containing YCplac111-*GAL10-TTI2* as the sole source of *TTI2* were grown in YP or synthetic dropout media containing 2% galactose except as otherwise indicated.

All strains in this study (Supplemental Material, Table S1) are derivatives of the wild-type yeast strains BY4741, BY4742, and BY4743 (Winzeler and Davis 1997). The *tti2* disruption strain CY6032 was generated in the diploid BY4743 strain by integrating a *TTI2*-Tn*10*luk*-MET5* cassette (described in *DNA constructs*). This strain was subsequently transformed with YCplac111-*DED1-TTI2* (*LEU2* centromeric plasmid), sporulated, and *URA3/LEU2* haploids selected to give rise to the *MATa* strain CY6049. The *URA3* was then eliminated by counter-selection with 5-fluoroorotic acid (5-FOA; Toronto Research Chemicals Inc.). YCplac33-*DED1-TTI2* (*URA3* centromeric plasmid) was transformed into CY6049, and YCplac111-*DED1-TTI2* lost after repeated growth on leucine containing plates to generate CY6070. This strain was subsequently transformed with YCplac111-*DED1-TTI2*, YCplac111*-DED1-tti2_L187P_*, and YCplac111*-DED1-tti2_Q276TAA_*, then plated on 5-FOA to lose YCplac33-*DED1-TTI2* and generate strains CY6857, CY6872, and CY6874, respectively. The related *MAT*α strain CY6963 was obtained by sporulation after crossing BY4742 with CY6070. CY6971 and CY6991 were made by transforming CY6070 with YCplac111-*GAL10-TTI2* and YEplac181-*GAL10-TTI2*, respectively, and in each strain the *DED1-TTI2URA3* plasmid was lost by plasmid shuffling on 5-FOA. To generate CY6973, the plasmid copy of *LEU2* was disrupted with *URA3* by integrating pLU12 (Cross 1997) digested with *Hpa*I and *Sal*I. CY7000 is a diploid strain containing a disruption of both genomic copies of *TTI2* made by mating CY6971 with CY6963, and subsequently losing the *URA3* on 5-FOA.

CY7086 expressing C-terminal Myc^9^-tagged Tti2 from its endogenous promoter was constructed by transforming BY4742 with the *Sph*I–*Sac*I fragment of pCB2890, and selecting for growth on medium lacking histidine. Integration of the Myc^9^-tag was confirmed by Western blot. To generate CY7172, pCB2911 was transformed into CY6971, repeatedly grown on leucine-containing plates depleted of uracil to lose YCplac111-*GAL10-TTI2*, then checked for leucine auxotrophy.

Flag^5^-tagged *TRA1* (CY5919) and Flag^5^-tagged *MEC1* (CY6194) strains have been previously described (Genereaux *et al.* 2012; DaSilva *et al.* 2013*)*. CY6999 and CY7030 were made by integration of the Flag^5^-tagged *TRA1* and *MEC1* containing cassette into CY6971, as described in Genereaux *et al.* (2012) and DaSilva *et al.* (2013). CY6415 containing a Flag^5^-tagged *TOR1* allele was made by integration of a *URA3*-containing cassette (described below) into BY4743 and CY7000.

Strains expressing *eGFP-TRA1* (CY5998 and CY7193) were made by integrating an *Sph*I–*Xba*I fragment of pCB2301 (as described in Genereaux *et al.* 2012). Similarly, *eGFP-MEC1* strains (CY6306 and CY7189) were made by integrating an *Sph*I–*Eco*RI fragment of pCB2395 (as described in DaSilva *et al.* 2013).

CY6857 and CY6872 were transformed with a two micron *URA3*-containing plasmid expressing *htt25Q* from the *GAL1* promoter (described in Duennwald *et al.* 2006) to generate CY7236 and CY7238, or *htt103Q* expressed from the *GAL1* promoter to create CY7237 and CY7239. For an empty vector control, YCplac33 was transformed into CY6872, resulting in CY7241.

Two micron *LEU2* plasmids containing *CDC37*, *HSP82*, *AHA1*, or *HSC82* expressed from the *GPD* promoter were transformed into CY6070 to generate strains CY7370, CY7371, CY7372, and CY7373, respectively, and into CY6973 to create strains CY7374, CY7375, CY7376, and CY7377, respectively. Two micron *URA3* plasmids expressing either *HSP42*, *HSP26*, or *HSP104* (described in Cashikar *et al.* 2005) were transformed into CY6857 to generate CY7323, CY7247, and CY7248, respectively, and into CY6971 to produce CY7324, CY7251, and CY7252, respectively. YCplac33 was transformed into CY6857 and CY6971 to give rise to empty vector control strains CY7245 and CY7249, respectively.

### DNA molecules

The *tti2* disruption cassette in pCB2312 was created in three steps. First, genomic DNA was used to PCR amplify a *Sal*I–*Bam*HI fragment at the 3′ end of *TTI2* extending into noncoding sequence using primers 6085-3 and 6085-4 (see Table S2 for a listing of oligonucleotides), which was cloned into pTZ18r. A 5′ fragment of *TTI2* extending into the *MET5* fragment was amplified using primers 6085-1 and 6085-2, and inserted as a *Bam*HI–*Eco*RI into the above plasmid. Tn*10*luk (Huisman *et al.* 1987) was then inserted into the *Bam*HI site of this molecule. Digestion with *Sal*I and *Eco*RI allowed insertion into yeast with selection for Ura^+^ transformants.

pCB2134 and pCB2319 express Myc^9^-tagged *TTI2* from the *DED1* promoter in the *LEU2* and *URA3* centromeric plasmids YCplac111 and YCplac33, respectively (Genereaux *et al.* 2012). The *GAL10* promoter was substituted into pCB2134 after cloning a PCR product using oligonucleotides 2764-1 and 2764-2 as a *Pst*I–*Hin*dIII fragment to generate pCB2844 (YCplac111-*GAL10-TTI2*). *GAL10-TTI2* from pCB2844 was inserted as a *Pst*I–*Sac*I fragment into YEplac181 to give pCB2862.

The *PHO5-lacZ* and *STRE-lacZ* fusion constructs in YCp87 have been previously described (Mutiu *et al.* 2007; Hoke *et al.* 2010). The *HSE-lacZ* plasmid has been described in Duennwald and Lindquist (2008).

pCB2890, the molecule to integrate a C-terminal Myc^9^-tag into Tti2, was constructed in a cassette using a base oligonucleotide synthesized by Life Technologies Inc. (see Figure S1 for sequence). This molecule was cloned as an *Sph*I–*Sac*I fragment into pTZ19r lacking a *Hin*dIII site. The *Sph*I site is at base pair 1076 relative to the *TTI2* translational start, followed by the TTI2 coding sequence to the C-terminus, with the Myc^9^-tag from YCPlac111-*DED1-TTI2* cloned as a *Hin*dIII–*Not****I*** fragment in front of the translational stop codon. The molecule contains a *Bam*HI site downstream of the translational stop into which *HIS3* was cloned, followed by additional downstream sequence and a *Sac*I site to allow integration into yeast as a *Sph*I-*Sac*I fragment. The *TTI2* promoter was PCR amplified using primers TD0569 and TD0570 and genomic DNA as template, cloned into YCplac33 as a *Sal*I-*Hin*dIII fragment in a triple ligation with *TTI2* from pCB2134 as a *Sal*I-*Sac*I fragment to generate pCB2911.

pCB2425, which contains a cassette to integrate Flag^5^-tagged *TOR1* as an *Sph*I–*Eco*RI fragment was adapted from pCB2134 used previously to tag *TRA1* (Genereaux *et al.* 2012). *TOR1*-specific sequences were added to this molecule as *Sph*I–*Hin*dIII and *Not*I–*Sal*I fragments after PCR with oligonucleotides 6496-1 and 6496, and 6496-3 and 6496-4. Digestion with *Sph*I and *Sal*I allows insertion into at *TOR1* after selection for Ura^+^ transformants.

*CDC37*, *HSP82*, *HSC82*, and *AHA1* were amplified by PCR (refer to Table S2 for oligonucleotide sequences) using genomic DNA as template and cloned into Gateway *GAL1* two micron destination vectors as described in Alberti *et al.* (2007).

The plasmids used for overexpressing Hsp26 and Hsp104 from the glyceraldehyde-3-phosphate dehydrogenase (GPD) promoter have been previously described (Cashikar *et al.* 2005). *HSP42*, along with its promoter (500 bp sequence upstream of translation start site), was cloned by PCR using primer TK7578 and TK7579 and genomic DNA as template. The PCR product was ligated into a *URA3* two micron plasmid (YEplac195) as a *Not*I–*Sac*I fragment.

### Screening for defective tti2 alleles

To generate strains CY6872 and CY6874, a library of randomly mutagenized *TTI2* alleles was constructed by PCR using *Taq* polymerase and primers 5693-1 and 5693-2, and inserting into YCplac111-*DED1-TTI2* as a *Not*I–*Sac*I fragment. The randomly mutated alleles were transformed into CY6070, and *TTI2* on the *URA3* plasmid was shuffled out by plating on 5-FOA. These strains were patched onto a YPD plate and screened for slow growth on a YPD plate containing 6% ethanol or when grown at 37°. Plasmids were recovered (Hoffman and Winston 1987) and transformed back into CY6070 to confirm the phenotype. Alleles resulting in slow growth were sequenced to identify mutations.

### Growth assays

Stress sensitivity assays were performed at 30° or 37° on YP plates containing 2% raffinose or glucose, and either 6% ethanol, 1 nM rapamycin (LC Laboratories), 0.1 M hydroxyurea (Sigma-Aldrich), 8 μg/ml Calcofluor white (Sigma-Aldrich), 10 mM caffeine (Sigma-Aldrich) or 1 M sodium chloride (EMD Chemicals Inc.). Strains were grown to stationary phase, their optical density at 600 nm normalized, and spotted in 10-fold serial dilutions onto each plate. Growth curves were performed in YP media with either 2% glucose, 2% galactose, or 2% raffinose as the carbon source after growth of a starter culture in YP containing 2% raffinose.

### Western blotting

Yeast strains were grown to midlogarithmic phase, then lysed with glass beads to harvest protein. Western blotting was performed using PVDF membranes and anti-Flag (M2; Sigma-Aldrich) and anti-Myc (9E10; Sigma-Aldrich) monoclonal antibodies as described by Mutiu *et al.* (2007) and Hoke *et al.* (2010). Anti-Hsp42 polyclonal antibody was kindly provided by Johannes Buchner (Technische Universität München), and was used as previously described (Haslbeck *et al.* 2004).

### Half-life of Tti2

Yeast strains CY6971 and BY4742 were grown to stationary phase in YP medium containing galactose, diluted 1:100 in the same medium, then grown to an optical density of 2.0 before adding 35 µg/ml of cycloheximide; 10^8^ cells were harvested before adding cycloheximide, and at 2, 4, 6, and 8 hr thereafter. Protein was extracted as described in von der Haar (2007) in the presence of protease inhibitors (1.0 mM phenylmethylsulfonyl fluoride, 5 mg/ml pepstatin, 1.0 mM benzamidine, 50 mg/ml trypsin inhibitor, and 5 mg/ml leupeptin), and the lysates were separated by SDS-PAGE and Western blotted with anti-Myc antibody. The bottom quarter of the gel was stained with Coomassie Brilliant Blue and shown as a control for equal loading.

### β-Galactosidase assays

LacZ reporter plasmids were assayed in strains CY6070 and CY6973. For the *PHO5-lacZ* reporter, strains were grown to stationary phase, washed three times with sterile water, diluted 20-fold in YPD medium depleted of phosphate, and grown for 8 hr. Cell densities were normalized and β-galactosidase units were determined using *o*-nitrophenol-β-d-galactosidase as substrate as described in Mutiu *et al.* (2007). Strains containing the *STRE-lacZ* reporter were diluted 1:20 from stationary culture, and grown in medium containing 2% glucose and 6% ethanol for 8 hr prior to the assay. When assaying the heat shock element, strains were grown to midlogarithmic phase in YPD, then heat shocked at 42° for 30 min. Strains containing *PGK-lacZ* were grown to stationary phase in medium with raffinose as the sole carbon source, then diluted 1:20 in YPD and grown for 8 hr.

### Fluorescence microscopy

Yeast strains CY5998, CY7193, CY6306, and CY7189 were grown to stationary phase in minimal medium lacking uracil, diluted 1:20 and grown to an optical density of 0.8 at 600 nm. Cells were concentrated 10-fold and 33258 Hoechst bisbenzimidazole dye (Sigma-Aldrich) was added to each culture at 1.0 µg/ml 1 hr prior to imaging. Cells were washed twice in 1 ml of sterile water and imaged using a Zeiss Axioskop 2 microscope driven by Image J 1.41 software (National Institutes of Health), and a Scion CFW Monochrome CCD Firewire camera (Scion, Frederick, MD) with bright field, DAPI, and GFP filter sets. Image J 1.48 software was used for quantifying GFP signal intensities. For each cell counted, the *elliptical* tool was used to trace each cell on bright field images, and the *measure* function was used to calculate signal intensity per unit area from GFP images. The nucleus was defined as the top 10% of pixels with the highest intensity values within the outlined cell from images taken with the DAPI filter, and was determined using the *threshold* function. This area was outlined using the *magic wand* tool and the traced area was used to measure GFP signal intensity in the nucleus. Background noise was subtracted from the whole cell and nuclear intensity values and was measured in the area adjacent to the cell. Percent intensity in the nucleus was calculated by dividing the background subtracted intensity values of the nucleus by the background-subtracted values of the whole cell. Percent nuclear intensity calculations represent an average of individual cell ratios (nuclear GFP intensity: whole cell GFP intensity) taken across 20 cells for each strain.

### Semidenaturing detergent-agarose gel electrophoresis (SDD-AGE)

Aggregation of Mec1 was analyzed using SDD-AGE as described in Kryndushkin *et al.* (2003) with the following modifications. Yeast strains CY6194 and CY7030 were grown to stationary phase in minimal medium lacking uracil and containing 2% raffinose. BY4742 was grown in YPD to stationary phase. All strains were diluted 1:20 in YPD and grown to midlogarithmic phase. Protein was extracted from cells by glass bead lysis in buffer containing 100 mM Tris, pH 7.5, 200 mM NaCl, 1.0 mM EDTA, 5% glycerol, 1.0 mM DTT, and protease inhibitors (1.0 mM phenylmethylsulfonyl fluoride, 5 mg/ml pepstatin, 1.0 mM benzamidine, 50 mg/ml trypsin inhibitor, and 5 mg/ml leupeptin). Protein lysates were separated on a 1.8% agarose gel then transferred to PVDF overnight by capillary transfer in Tris-buffered saline pH 7.5. The PVDF membrane was Western blotted using anti-Flag primary and mouse secondary antibodies as described previously (Mutiu *et al.* 2007; Hoke *et al.* 2010). Ponceau S (Sigma-Aldrich) was used to stain the membrane after capillary transfer to document equal loading.

### Data availability

All yeast strains and plasmids are available upon request. The authors state that all data necessary for confirming the conclusions presented in the article are represented fully within the article.

## Results

### Identification of stress-sensitive tti2 alleles

**Figure 1:**
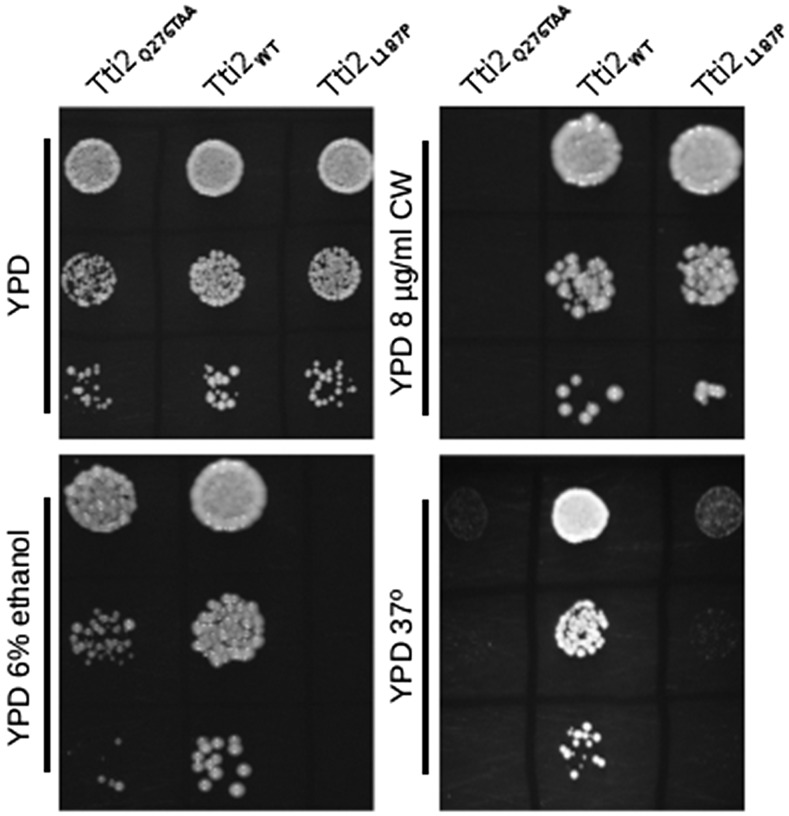
Selection of randomly created tti2 mutations that cause slow growth. CY6070 (*TTI2*), CY6872 (*tti2_L187P_*), and CY6874 (*tti2_Q276TAA_*) were grown to saturation in YPD medium then spotted in 10-fold serial dilutions onto YPD, YPD containing 6% ethanol, or 8 μg/ml Calcofluor white, and grown at 30°, and on YPD grown at 37°.

To initiate studies into the structure/function relationships of Tti2, we selected for randomly created mutations that confer slow growth under stress conditions. A library containing random mutations in *TTI2* generated by PCR with *Taq* polymerase was transformed into CY6070, a strain with the genomic copy of *TTI2* deleted, and with *TTI2* on a *URA3*-containing plasmid. After plasmid shuffling on 5-FOA, we screened colonies for slow growth on YPD plates containing 6% ethanol and/or when grown at 37°. Two strains were identified. The plasmids from each strain were then sequenced and the phenotype confirmed after retransformation. One *tti2* allele contained a mutation converting leucine 187 to proline; the second allele, *tti2_Q276TAA_*, contained an ochre mutation at codon 276. As shown in Figure 1, the L187P mutation causes a slight reduction in growth on YPD at 30°, and more severely reduced growth on medium containing 6% ethanol, and at 37°. The *tti2_Q276TAA_* allele resulted in slow growth under each condition, with severely reduced growth on medium containing the cell wall binding compound Calcofluor white and at 37°.

### Low levels of Tti2 support viability but cause slow growth

**Figure 2:**
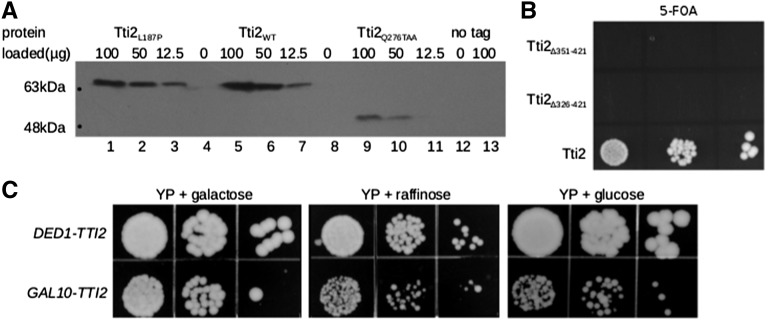
Characterization of Tti2 mutations. (A) Yeast strains CY6872 (lanes 1, 2, and 3), CY6070 (lanes 5, 6, and 7), CY6874 (lanes 9, 10, and 11), and BY4742 (lane 13), were grown to stationary phase in minimal medium, diluted 1:20 in YPD and grown for 8 hr at 30°. Protein was harvested by grinding with glass beads, and the indicated amount of lysate separated by SDS-PAGE (8%), and Western blotted with anti-Myc antibody. (B) CY6070 was transformed with plasmids containing full length *TTI2*, or derivatives with C-terminal deletions to residues 350 (Δ351–421) or 325 (Δ326–421). Individual transformants were grown in YPD and spotted serially on a minimal plate containing 5-FOA. (C) Strains CY6070 (*DED1-TTI2*) and CY6971 (*GAL10-TTI2*) were grown to stationary phase, then spotted in 10-fold serial dilutions onto YP plates containing 2% galactose, raffinose, or glucose, and grown for 3 d at 30°.

As the *tti2* alleles were Myc^9^-tagged, we were able to examine their expression by Western blotting (Figure 2A). Tti2_L187P_ is expressed at a level approximately two-fold less than the wild-type protein. *tti2_Q276TAA_* is expressed at a reduced level, and, as expected, is truncated. The ability of *tti2_Q276TAA_* to support viability may indicate that the C-terminal sequence of Tti2 is not essential for function, or that the ochre mutation is read-through (or skipped) at a low frequency, which is sufficient to support viability. To test if the C-terminus of Tti2 is essential, we used plasmid shuffling to examine whether C-terminal truncations to residue 356 and 321 support viability. As shown in Figure 2B, neither truncation allele supports viability. We therefore conclude that C-terminal sequences are essential, and hypothesize that *tti2_Q276TAA_* supports viability due to a low level of read-through of the ochre mutation.

To test the effect of reducing *TTI2* expression, we placed it under the control of the *GAL10* promoter on a *LEU2* centromeric plasmid in a strain lacking the genomic copy (CY6971). Comparisons were made between *TTI2* expressed from the *DED1* and the *GAL10* promoter. When grown on galactose-containing medium, *GAL10-TTI2* supported growth at a rate comparable to *DED1-TTI2* (Figure 2C). To test for growth on raffinose and glucose-containing plates, conditions that repress *GAL10* transcription, cells were first grown in liquid culture containing the respective carbon source. Similar to what was observed with *tti2_Q276TAA_*, *GAL10-TTI2* supported viability but showed reduced growth when cells were grown with these carbon sources. These results were reproduced in liquid medium, where growth of the *GAL10-TTI2* strain in glucose and raffinose decreased the doubling time of cells in logarithmic phase by approximately three- and 1.5-fold (relative to *DED1-TTI2*), respectively (Table 1).

**Table 1:** Doubling times (hours) of strains during logarithmic growth phase in YP media containing the indicated carbon source

Strain	Galactose	Raffinose	Glucose
CY6070 (*DED1-TTI2*)	2.0 +/− 0.1	2.8 +/− 0.1	1.50 +/− 0.01
CY6973 (*GAL10-TTI2*)	2.5 +/− 0.1	4.1 +/− 0.1	4.4 +/− 0.4

**Figure 3:**
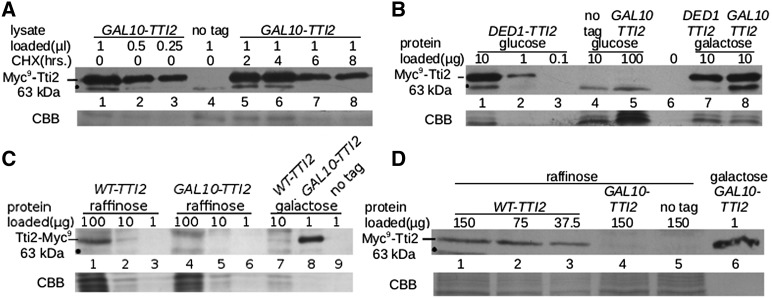
Depleting Tti2 below endogenous levels supports viability. (A) Yeast strains CY6971 and BY4742 were grown to saturation in YP medium containing galactose, then diluted 1:100 in the same medium, and grown for 16 hr to an optical density of 2.0 at 600 nm; 10^8^ cells were then harvested before blocking translation and 2, 4, 6, and 8 hr after adding 35 µg/ml of cycloheximide. For each time point, cell pellets were washed with lysis buffer then immediately stored at –80°. Total protein was extracted from each cell pellet using an optimized extraction protocol (von der Haar 2007) and in the presence of protease inhibitors. A 1.0-µl aliquot of lysate was loaded per time point and separated by SDS-PAGE. Tti2 was detected by Western blotting with anti-Myc antibody, and the bottom of the gel was stained with Coomassie Brilliant Blue for a loading control. (B) *GAL10-TTI2* expression in glucose-containing medium. Yeast strain CY6070 (lanes 1, 2, and 3), CY6971 (lanes 5 and 8), and BY4742 (no tag control; lane 4) were grown to stationary phase in glucose-containing YP, diluted 1:20 in fresh medium and grown for 8 hr before harvesting. Protein was extracted with glass beads and the indicated amounts separated by SDS-PAGE and Western blotted with an anti-Myc antibody to detect tagged Tti2. Lane numbers are listed between the blot and the bottom of the gel, which was stained with Coomassie Brilliant Blue for a loading control (CBB). Lane 6 was left open to separate galactose control lanes. (C) CY7086 (genomically encoded C-terminal Myc^9^-tagged *TTI2*, *WT-TTI2*; lanes 1, 2, 3, and 7), CY6971 (*GAL10-TTI2*; lanes 4, 5, 6, and 8), and BY4742 (no tag; lane 9) were grown to saturation in YP media containing either raffinose or galactose, then diluted 1:20 in the same medium and grown for 8 hr. Protein was extracted by bead lysis and the indicated amounts separated by SDS-PAGE and Western blotted as described above. The bottom portion of the gel was stained with Coomassie Brilliant Blue. (D) CY6971, CY7172 (YCplac33-*TTI2-TTI2*), and BY4742 were grown to stationary in media depleted of leucine and containing either raffinose or galactose, diluted 1:20 in the same medium, and grown for 8 hr before harvesting. Protein extraction and Western blotting was performed as in (B).

To ensure that the observed doubling times were not influenced by excess Tti2 remaining in the cells from their prior growth in galactose, we determined protein half-life. The *tti2* disruption strain containing *GAL10-TTI2* (CY6971) was grown to midlogarithmic phase with galactose as the carbon source, and translation was blocked with cycloheximide. Tti2 levels decreased approximately two-fold after 6 hr (Figure 3A; cf. lanes 2 and 7). We conclude that by growing cells in raffinose or glucose containing media over 2 d, we can deplete the cellular levels of Tti2 and assess the effect on growth.

We then examined the steady state expression of Tti2 in different media by Western blotting. The amount of Tti2 was similar when expressed from the *GAL10* or *DED1* promoter when cells were grown in galactose-containing medium (Figure S2A). In contrast, Tti2 was not detected in 100 µg of protein extract when the *GAL10-TTI2* allele was expressed in glucose (Figure 3B; lane 5) or raffinose media (Figure S2B; lane 5). In these same conditions, *DED1-TTI2* expression was detected in 1 μg of extract (Figure 3B and Figure S2B; lane 2). To provide an indication of the relative expression of *GAL10-TTI2* in raffinose and glucose media, we engineered the constructs in the two micron plasmid YEplac181. The level of expression from the two micron plasmid supported robust growth on glucose-containing plates (Figure S3A). *GAL10* expressed Tti2 from the two micron plasmid was detected in raffinose and glucose-containing media, with the expression in raffinose being approximately five-fold higher than in glucose (Figure S3B; cf. lanes 4 and 7), a fold-change consistent with what we routinely observe with LacZ-fusions to the *GAL10* promoter.

Two approaches were taken to demonstrate that depleting Tti2 using the *GAL10* promoter results in expression below endogenous levels. First, the expression of an integrated C-terminally Myc^9^-tagged *tti2* allele was compared to *GAL10-TTI2* expression from a centromeric plasmid in raffinose-containing medium. Again, Tti2 expressed from the *GAL10* promoter was not detected with 100 μg of protein loaded (Figure 3C, lane 4). In comparison the endogenous allele allowed detection at 10 μg (lane 2), suggesting a minimum difference of 10-fold in raffinose and by extrapolation 50-fold in glucose medium. Second, we analyzed Tti2 expression regulated by the *TTI2* promoter on a centromeric plasmid. Tti2 was detected when expressed from its promoter at 37.5 µg of protein extract (Figure 3D, lane 3), while expression from the *GAL10* promoter in raffinose was undetectable in 150 µg of protein loaded (lane 4). Taken together, these results indicate that expression of Tti2 below endogenous levels supports viability but lead to slow growth.

### Depleting Tti2 levels causes sensitivity to stress conditions

**Figure 4:**
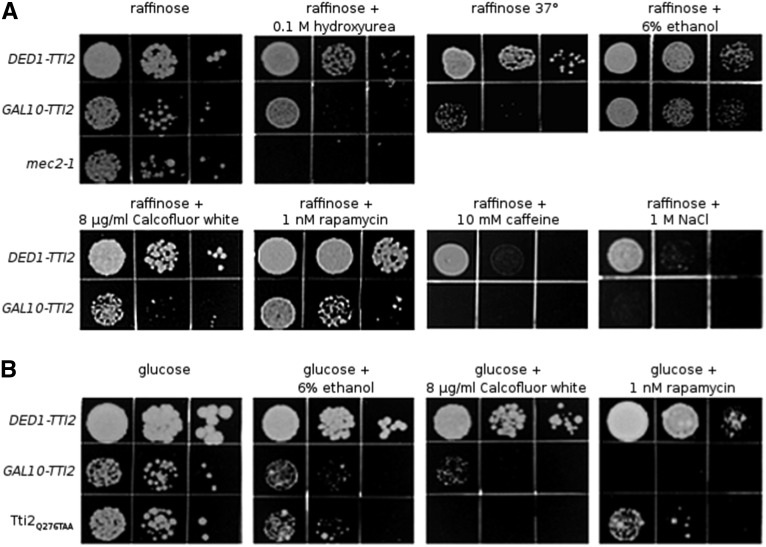
Stress sensitive phenotypes caused by reduced expression of *TTI2*. (A) CY6070 (*DED1-TTI2*), CY6971 (*GAL10-TTI2*), and *mec2-1* (included as a control for sensitivity to hydroxyurea; Weinert *et al.* 1994), were grown to stationary phase in YP medium containing raffinose then 10-fold serial dilutions spotted onto YP plates containing raffinose, or with raffinose, and either 0.1 M hydroxyurea, 6% ethanol, 8 μg/ml Calcofluor white, 1.0 nM rapamycin, or 1 M NaCl and grown for 3 d at 30° or 37°. (B) CY6070, CY6971, and CY6874 (*tti2_Q276TAA_*) were grown to stationary phase in YPD medium then 10-fold serial dilutions spotted onto YPD plates, or YPD with either 6% ethanol, 8 μg/ml Calcofluor white, 1.0 nM rapamycin, or 10 mM caffeine, and grown at 30° for 3 days.

Strains containing YCplac111-*GAL10-TTI2* were analyzed to determine whether depleting Tti2 would affect growth under stress conditions, possibly by regulating PIKK expression. Serial dilutions of the *GAL10-TTI2*- and *DED1-TTI2*-containing strains were spotted onto plates with raffinose as the carbon source, and also on raffinose containing either hydroxyurea (ribonucleotide reductase inhibitor), ethanol, Calcofluor white (cell wall integrity), rapamycin (mTOR inhibitor), caffeine, and sodium chloride (osmotic stress) (Figure 4A). Growth at 37° was also examined. Slow growth was observed in all of these stress conditions. For conditions that had more moderate effects on growth, we repeated the assay on plates with glucose as the carbon source. In each case (ethanol, Calcofluor white, and rapamycin) this resulted in a more severe growth defect (Figure 4B). The pattern resembled that seen for the *tti2_Q276TAA_* allele; however, the *GAL10-TTI2* strain was more sensitive to rapamycin and slightly less sensitive to Calcofluor white. Taken together, these results indicate that low levels of Tti2 cause stress sensitivity to conditions that perturb pathways regulated by PIKK proteins, including the DNA damage response, cell wall integrity pathway, nutrient limitation, and osmoregulation.

### Depleting Tti2 reduces PIKK steady state levels

**Figure 5:**
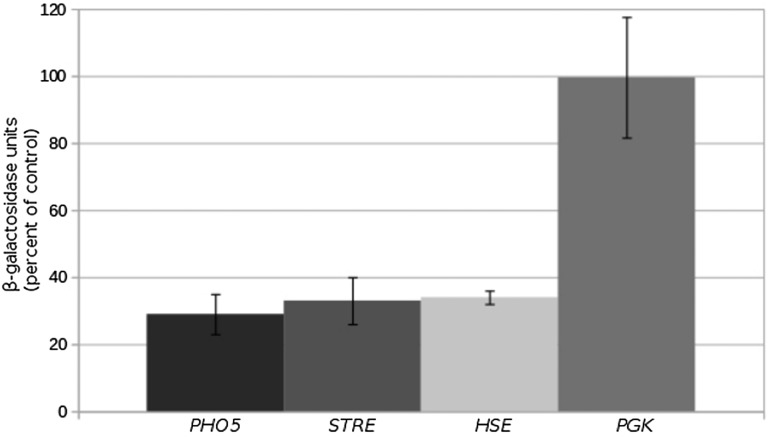
Low levels of Tti2 affect expression of Tra1-regulated promoters. Yeast strains CY6070 (*DED1-TTI2*) and CY6973 (*GAL10-TTI2*) were transformed with either a *PHO5-lacZ* fusion, the stress response element (STRE)-regulated *lacZ* fusion, the heat shock element (HSE)-regulated *lacZ* fusion, or *PGK-lacZ* on a *LEU2* centromeric plasmid. For the *PHO5* promoter, strains were grown to stationary phase in raffinose medium depleted of leucine, cells were washed three times with water before being diluted 1:20 in YPD medium depleted of phosphate and grown for 8 hr. Cell densities were normalized and β-galactosidase activity determined. The β-galactosidase units are the average of three replicates, with the SD shown by error bars. Average enzyme units are compared as the percentage of *GAL10-TTI2* to the *DED1-TTI2* strain. Strains containing *STRE-lacZ* fusions were grown to stationary phase in raffinose medium depleted of leucine, diluted 1:20 in YPD medium containing 6% ethanol and grown for 16 hr. *HSE-lacZ* expression was analyzed in strains grown to stationary phase in raffinose medium depleted of leucine, diluted 1:20 in YPD medium, grown for 8 hr then heat shocked at 42° for 30 min. *PGK-lacZ* was analyzed after growing cells to stationary phase in raffinose medium depleted of leucine, diluting 1:20 in YPD and growing for 8 hr.

The nature of the stress-related growth defects following reduced levels of Tti2 suggested that PIKK proteins are affected. We previously found that reduced levels of Tra1 alter the expression of genes involved in stress response (Hoke *et al.* 2010). To address whether Tra1 was affected by depleting Tti2, we analyzed transcription regulated by a stress response element (STRE), a heat shock element (HSE), and the *PHO5* promoter. Tti2 levels were depleted in the *GAL10-TTI2* strain by growing in medium containing glucose, and β-galactosidase activity was compared to that of the *DED1-TTI2*-containing strain. The *STRE* promoter was activated by growth in medium containing 6% ethanol. The *PHO5* promoter was activated by growth in phosphate-depleted medium, and the heat shock element by growth at 42°. Activity of the *PGK* promoter was analyzed under steady state conditions and used as a negative control. As shown in Figure 5, expression decreased approximately three-fold in the *GAL10-TTI2* strain for each promoter except *PGK*, suggesting that Tti2 is required for transcription at Tra1-regulated promoters.

**Figure 6:**
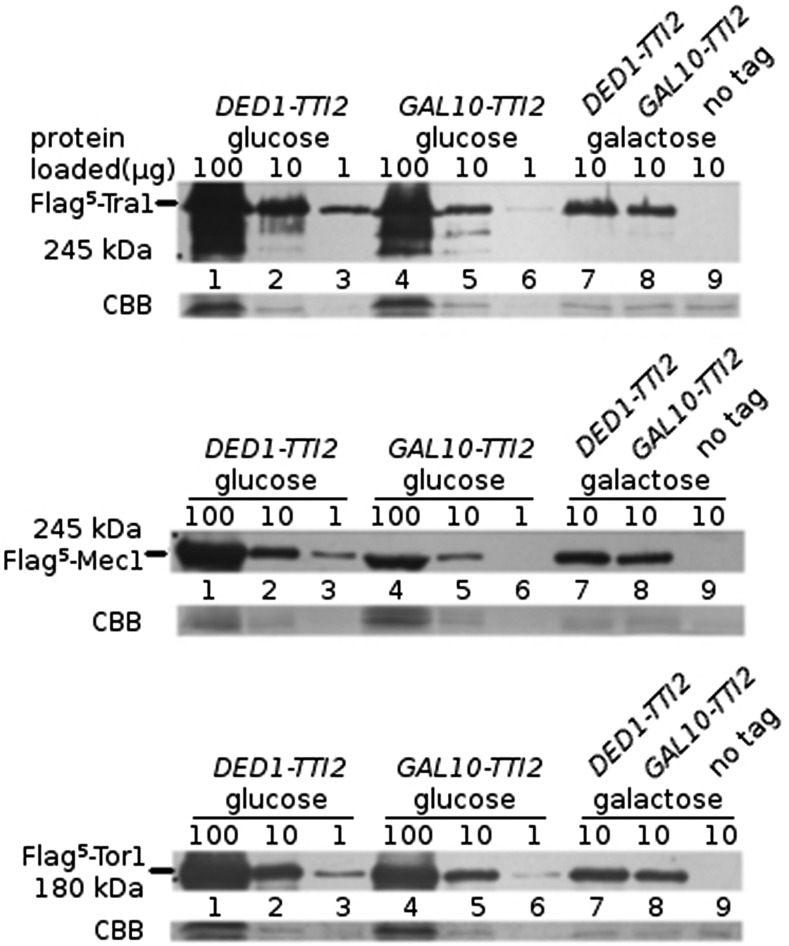
Expression of Tra1, Mec1, and Tor1 are reduced when Tti2 is depleted. (A) Yeast strains CY5919 (*Flag^5^-TRA1 TTI2*; lanes 1, 2, 3, and 7), CY6999 (*Flag^5^-TRA1 tti2*::Tn10*luk GAL10-TTI2*; lanes 4, 5, 6, and 8), and BY4742 (wild type no tag; lane 9) were grown to stationary phase in media containing glucose or galactose (indicated above each blot), diluted 1:20, and grown for 8 hr before extracting protein by bead lysis. The indicated amount of protein (in micrograms) was separated by SDS-PAGE. Anti-Flag (M2) antibody was used to Western blot the top portion of each gel, and the bottom was stained with Coomassie Brilliant Blue (CBB). (B) Protein lysates from strains CY6194 (*Flag^5^-MEC1 TTI2*; lanes 1, 2, 3, and 7), CY7030 (*Flag^5^-MEC1 tti2*::Tn10*luk GAL10-TTI2*; lanes 4, 5, 6, and 8), and BY4742 (wild type no tag; lane 9) were prepared and Western blotted as described in (A). (C) Protein lysates from strains CY6415 (*Flag^5^-TOR1/TOR1 TTI2/TTI2*; lanes 1, 2, 3 and 7), CY7035 (*Flag^5^-TOR1/TOR1 tti2*::Tn10*luk*/ *tti2*::Tn10*luk GAL10-TTI2*; lanes 4, 5, 6, and 8), and BY4742 (wild type no tag; lane 9) were prepared and Western blotted as described in (A).

To directly test if reduced levels of Tti2 alter the expression of PIKK proteins, we examined the levels of Flag^5^-Tra1, Mec1 and Tor1 in cells containing *GAL10-TTI2*, comparing to a strain expressing *TTI2* from the *DED1* promoter. Tra1 expression was similar for cells grown in galactose (Figure 6A, lanes 7 and 8), but reduced approximately eight-fold for cells grown in glucose (for example, cf. lanes 3 and 6 in Figure 6A). Mec1 expression (Figure 6B) was similarly reduced in the *GAL10-TTI2* strain when cells were grown in glucose (densitometry indicated an 8.9-fold change). Tor1 expression was affected to a somewhat lesser extent compared to Tra1 and Mec1 after depleting Tti2, having an approximate 2.5-fold decrease in levels (Figure 6C).

### Mislocalization of Tra1 and Mec1 in the absence of Tti2

**Figure 7:**
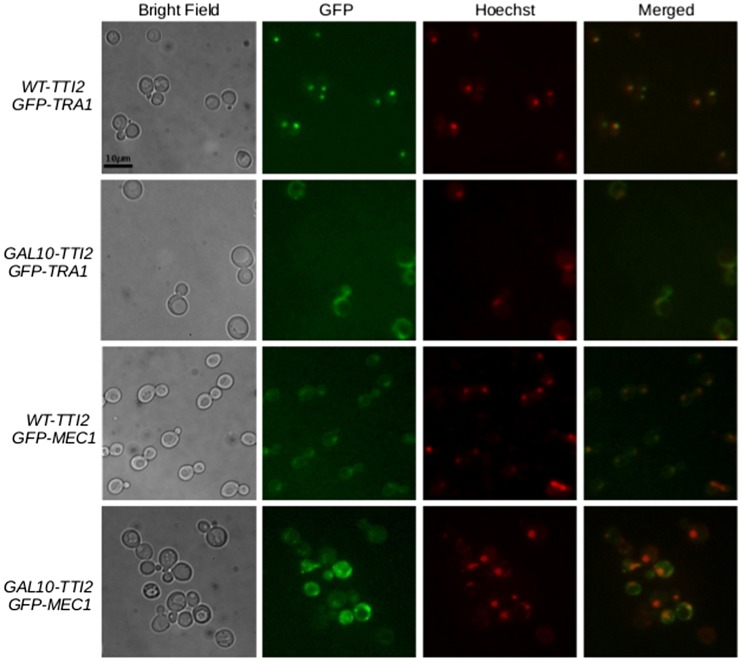
Localization of Tra1 and Mec1 upon depleting Tti2. Strains CY5998 (*eGFP-TRA1 WT-TTI2*), CY7193 (*eGFP-TRA1 GAL10-TTI2*), CY6306 (*eGFP-MEC1 WT-TTI2*), and CY7189 (*eGFP-MEC1 GAL10-TTI2*) were grown to stationary phase in synthetic complete medium containing raffinose, diluted 1:20 in synthetic complete medium containing glucose, grown to midlogarithmic phase, and stained with Hoechst stain. Cells were harvested and washed twice in sterile water, then imaged using fluorescence microscopy.

Tra1 and Mec1 normally localize to the nucleus; however, when the stability of each protein is compromised by mutations or truncations at the C-terminus, mislocalization to the cytoplasm occurs (Genereaux *et al.* 2012; DaSilva *et al.* 2013). Since the absence of Tti2 may affect the stability or folding of PIKKs, we determined if depleting Tti2 levels causes mislocalization of Tra1 and Mec1. We integrated an eGFP tag at the N-terminus of Tra1 and Mec1 into a wild-type strain, and in a strain expressing *TTI2* from the *GAL10* promoter. Upon depleting Tti2 levels, both Tra1 and Mec1 mislocalized to foci outside the nucleus. eGFP signal was also dispersed throughout the cytoplasm (Figure 7). When grown in minimal medium containing glucose, 38% of the fluorescent signal was localized to the nucleus in the eGFP-Tra1 strain expressing abundant Tti2, whereas 18% was nuclear when depleting Tti2. For eGFP-Mec1, 26% of the signal intensity was detected in the nucleus in wild-type cells compared to 14% when Tti2 levels were depleted. These results indicate that low levels of Tti2 cause mislocalization of Tra1 and Mec1 into foci and other areas throughout the cytoplasm.

We then examined if the cytoplasmic foci of eGFP-Mec1 seen upon depleting Tti2 resulted from the formation of Mec1 aggregates within the cell. To detect protein aggregation, we performed SDD-AGE, comparing the migration of Flag-tagged Mec1 from strains having either wild type or depleted levels of Tti2. The same molecular weight product for Flag-Mec1 was detected for both strains, indicating no change in protein aggregation (Figure S4). A protein product of a Huntington’s disease allele was used as a positive control for aggregation under the same semidenaturing conditions.

### Overexpressing Hsp90 chaperones and cochaperones is lethal when Tti2 is depleted

**Figure 8:**
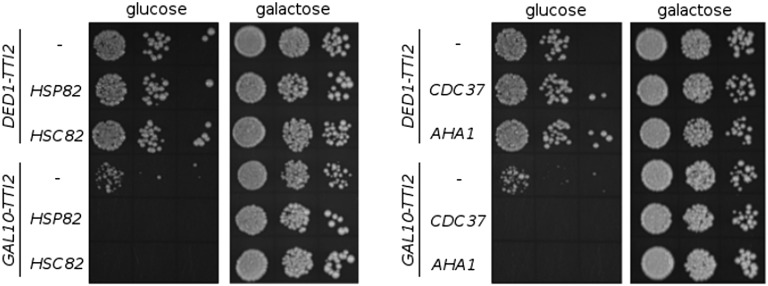
Overexpression of Hsp90 chaperones and cochaperones result in synthetic lethality in strains depleted for Tti2. Yeast strains CY7245 (*DED1-TTI2*), CY7249 (*GAL10-TTI2)*, CY7370 (*DED1-TTI2 GPD-CDC37*), CY7371 (*DED1-TTI2 GPD-HSP82*), CY7372 (*DED1-TTI2 GPD-AHA1*), CY7373 (*DED1-TTI2 GPD-HSC82*), CY7374 (*GAL10-TTI2 GPD-CDC37*), CY7375 (*GAL10-TTI2 GPD-HSP82*), CY7376 (*GAL10-TTI2 GPD-AHA1*), and CY7377 (*GAL10-TTI2 GPD-HSC82*) were grown to stationary phase in minimal medium containing 2% raffinose and lacking leucine. Cell densities were normalized then cells spotted in 10-fold serial dilutions onto both YPD and YP-galactose plates and grown at 30° for 2 d.

Tti2, along with Tel2 and Tti1, share interactions with Hsp90, and inhibiting Hsp90 prevents the association of Tel2 with PIKK proteins (Takai *et al.* 2010). We tested if Hsp90 overexpression could rescue slow growth caused by Tti2 depletion, possibly through an increase in the recruitment of Tti2 to PIKK peptides. We expressed Hsp90 chaperones Hsp82 and Hsc82, and cochaperones Cdc37 and Aha1, from multicopy plasmids in *tti2* disruption strains containing either *DED1-TTI2* or *GAL10-TTI2*. Interestingly, when Tti2 was depleted by growing the *GAL10-TTI2* cells in glucose-containing medium, overexpression of each chaperone or cochaperone resulted in synthetic lethality (Figure 8). This effect depended on depleting Tti2 levels as the *GAL10-TTI2* strains were viable on galactose-containing plates (Figure 8). Together with the results from Takai *et al.* (2010), these findings suggest that altered stoichiometry of protein interactions between Hsp90 chaperones, Hsp90 cochaperones, and TTT complex members is detrimental to the cell, possibly through an effect on PIKK assembly.

### Tti2 is required for stress responses

**Figure 9:**
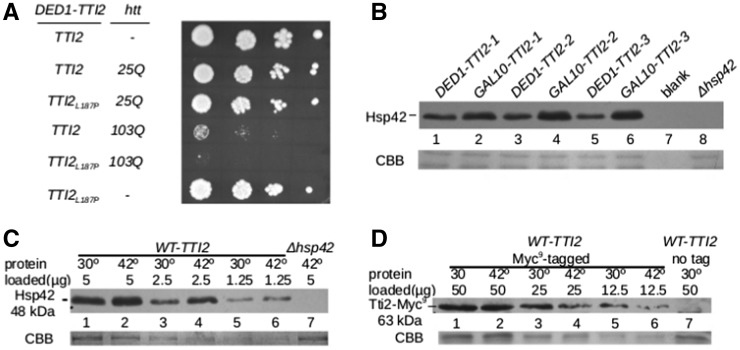
Tti2 is required for stress response. (A) Yeast strains CY7245 (*DED1-TTI2* YCplac33), CY7236 (*DED1-TTI2 GAL1-htt25Q*), CY7237 (*DED1-tti2_L187P_ GAL1-htt25Q*), CY7238 (*DED1-TTI2 GAL1-htt103Q*), CY7239 (*DED1-tti2_L187P_ GAL1-htt103Q*), and CY7241 (*DED1-tti2_L187P_* YCplac33) were grown to stationary phase in minimal medium containing 2% galactose and lacking uracil. Cell densities were normalized then cells were spotted in 10-fold serial dilutions onto a YP plate containing galactose, and grown at 32° for 2 d. (B) CY6070, C6973, and a Δ*hsp42* strain were grown to stationary phase in YP medium containing raffinose, diluted 1:20 in YPD, grown for 8 hr at 30° then harvested. Protein was extracted by bead lysis, and 10 µg of protein loaded for each sample. Western blotting was performed with anti-Hsp42 antibody. Lane 7 was left blank to avoid overflow into the Δhsp42 lane, and the bottom of the gel was stained with CBB as a loading control. (C) Yeast strain CY7086 (*WT-TTI2*) and BY4742 (no tag control) were grown to stationary phase in YPD, diluted 1:20, grown for 8 hr then heat shocked at 42° for 30 min. Protein extraction and Western blotting were done as described in (B), except with anti-Myc antibodies. The bottom of the gel was stained with CBB, and shown as a loading control. (D) The same CY7086 cell lysate from (C) was separated a second time by SDS-PAGE. Protein lysates were then Western blotted with anti-Hsp42 antibody. Protein extract from a Δhsp42 strain (Cashikar *et al.* 2005) was used as a negative control.

Considering the proposed role of Tti2 as a cochaperone, and that depleted or mutant Tti2 strains grow slowly at 37°, we hypothesized that Tti2 plays a role in responding to protein stress and managing misfolded proteins. To test this, we examined if exon 1 of the human Huntington gene containing a 103 residue polyQ (polyglutamine) sequence caused synthetic slow growth when expressed in the Tti2_L187P_-containing strain. The 103 residue polyQ sequence caused slow growth in both wild-type *TTI2* and *tti2-L187P* strains when expressed from the *GAL10* promoter on a two micron (Figure 9A) or centromeric plasmid (Figure S5), whereas a 25-residue polyQ sequence did not. Growth was more severely compromised in the *tti2_L187P_* background, suggesting a role for Tti2 in responding to protein stress. To determine whether reduced Tti2 function induces a heat shock response, we analyzed the expression of the heat inducible Hsp42 (Haslbeck *et al.* 2004) in strains containing *DED1-TTI2* or *GAL10-TTI2* when grown at 30° in glucose-containing medium. Steady state levels of Hsp42 were increased 3.8-fold when Tti2 was depleted (Figure 9B; average signal intensity was calculated using lanes 1, 3, and 5 for *DED1-TTI2*, and lanes 2, 4, and 6 for *GAL10-TTI2*). However, as shown in Figure 9C, Tti2 is not heat inducible, with the expression of endogenous Tti2 slightly decreased after shifting yeast strain CY7086 (*WT-TTI2-MYC^9^*) from 30° to 42° for 30 min, conditions in which Hsp42 was induced (Figure 9D). These results suggest that Tti2 does not play a general role in the heat shock response; however, depleting Tti2 results in protein stress.

As Hsp42 levels increase in the Tti2-depleted strain, we examined whether its overexpression, or overexpression of Hsp26 or Hsp104, could suppress slow growth and stress sensitivity caused by low levels of Tti2. Each of these heat shock proteins has been characterized for their role as molecular chaperones that bind unfolded or misfolded proteins, preventing or disrupting aggregation and mediating protein reactivation (Parsell *et al.* 1994; Haslbeck *et al.* 1999, 2004; Stromer *et al.* 2003). Hsp26, Hsp42, and Hsp104 were expressed from multicopy plasmids in *tti2* disruption strains containing either *DED1-TTI2* or *GAL10-TTI2*. Their overexpression did not result in the synthetic slow growth observed with overexpression of the Hsp90 chaperones and cochaperones, but also did not suppress slow growth caused by depleting Tti2 on any of the conditions tested (Calcofluor white, hydroxyurea, ethanol, and growth at 37°) (Figure S6).

## Discussion

Tti2 was initially characterized through its interaction with Tel2 and Tti1 in mammalian cells and *S. pombe* (Shevchenko *et al.* 2008; Hořejší *et al.* 2010; Takai *et al.* 2010). The studies in mammalian cells identified a role for Tel2 in the cellular level of PIKK proteins. The association of Tel2 with nascent PIKK peptides, and interactions of Tti2, Tel2, and Tti1 with chaperones and cochaperones including, Hsp70, Hsp90, Hsp40, and R2TP subunits (Hořejší *et al.* 2010; Takai *et al.* 2010), suggested they were involved in the biosynthesis of members of the PIKK protein family. In *S. cerevisiae*, mutation of *tel2* results in shortened telomeres (Lustig and Petes 1986), a telomere position effect (Runge and Zakian 1996), and defects in DNA repair (Anderson *et al.* 2008). This is likely due to reduced levels of Tel1 (Anderson *et al.* 2008), an effect also observed with mutation of *tti2* (Stirling *et al.* 2011). The effect of individual *S. cerevisiae* TTT proteins on cellular phenotypes, and on the PIKK family members has not been well characterized. This is likely in part because gene knockouts are inviable, and due to the limited availability of conditional alleles.

Our screen for alleles of *tti2* that resulted in growth defects identified two alleles, *tti2_L187P_* and *tti2_Q276TAA_*. We have found that with more extensive mutagenesis, additional alleles can be obtained with multiple mutations. The protein is predicted to be rich in alpha helices, and L187 is found within a predicted helix. The proline mutation may destabilize this helix, leading to partial loss of function. We have also analyzed numerous N- and C-terminal truncations of Tti2, and found that only short truncations at the C-terminus are viable. We were therefore initially surprised to see that the *tti2_Q276TAA_* allele supported viability. Although we do not see full-length protein by Western blotting, some read through is likely because ochre mutations are read through by tRNA_Gln_ (Pure *et al.* 1985; Edelman and Culbertson 1991). Such read through is enhanced if the context of the stop codon is not optimal (Brown *et al.* 1993). This has been observed previously for nonsense mutations (Kopczynski *et al.* 1992), and for native stop codons, and may be a regulated event (von der Haar and Tuite 2007).

The *GAL10*-expressed *TTI2* allele confirmed that low level expression of Tti2 is sufficient for viability. Further characterization revealed that the reduced expression leads to slow growth in many stress conditions. Many of these phenotypes are shared with mutations of PIKK encoding genes (*e.g.*, sensitivity to rapamycin, Calcofluor white, ethanol, hydroxyurea, and caffeine; see Table 2 for a list of the phenotypes examined). In addition, expression of Tra1-regulated genes decreases upon Tti2 depletion. This further suggests that Tra1 is affected by depleting Tti2, as we have found hypomorphic alleles of *TRA1* have these same characteristics (Hoke *et al.* 2010). Indeed the protein levels of epitope-tagged versions of Tra1, Mec1, and Tor1 were reduced in response to Tti2 depletion, with Tra1 and Mec1 steady state levels being decreased approximately eight-fold. Together with the result of Stirling *et al.* (2011) that Tel1 is reduced in a conditional *tti2* strain, our findings suggest that Tti2 is required for the expression of all of the PIKK proteins. The ability of Tti2 to influence the PIKK proteins places it at a strategic position to regulate cell function. The PIKK proteins are themselves regulators of gene expression, chromosomal stability, DNA damage response, and protein metabolism. Factors that influence Tti2 will thus have a profound effect on cell survival.

**Table 2:** Growth rates of the *GAL10-TTI2* strain (CY6971) under different stress conditions

Condition	Galactose	Raffinose	Glucose
6% ethanol	++	++	+
8 µg/ml Calcofluor white	++	+	+/−
0.1 M hydroxyurea	++	+/−	+/−
37°	++	+/−	+/−
1 M NaCl	++	+	+
1 nM rapamycin	++	++	+/−
10 mM caffeine	++	+	+

++, wild-type growth; +, slow growth; +/−, severe slow growth.

Our results support a role for Tti2 in protein biosynthesis, though perhaps more complex than acting exclusively in protein folding. As expected for a chaperone, Tra1, Mec1, and, to a lesser extent, Tor1 levels, are diminished upon Tti2 depletion. Nuclear localization of Tra1 and Mec1 is reduced and cytoplasmic foci apparent, potentially suggesting that Tti2 has a role in protein trafficking. The nature of the Mec1 and Tra1 foci is unclear, but they do not appear to result from aggregation. Consistent with Tti2 being required for proteostasis, its depletion induces expression of Hsp42, and the stress sensitive *tti2_L187P_* allele causes synthetic slow growth with overexpression of the Huntington gene PolyQ repeat sequences.

The TTT complex is associated functionally with Hsp90 and its cochaperones. Takai *et al.* (2010) demonstrated that the maturation of PIKK complexes is disrupted by depleting Tel2 or by inhibiting Hsp90. Furthermore, Tel2 links the R2TP/prefoldin like complex with Hsp90 and the PIKKs (Hořejší *et al.* 2010; Takai *et al.* 2010; Pal *et al.* 2014). Tti2 shares Hsp90 and R2TP interactions with Tel2, and the binding of Tel2 with Tti1-Tti2 is important for PIKK levels (Hurov *et al.* 2010; Takai *et al.* 2010; Stirling *et al.* 2011). These findings suggest that the toxicity we observe when Hsp90 or its cochaperones are overexpressed in strains depleted for Tti2, is due to a role for Tti2 with Tel2 and Tti1 in mediating the association of PIKK proteins with Hsp90 and R2TP/prefoldin. We favor the idea that the toxicity is caused by Hsp90 or the cochaperones sequestering the low level of Tti2 into nonproductive complexes, though we cannot exclude that Tti2 normally functions to negatively inhibit what are detrimental effects of the Hsp90 chaperones.

The finding that cells can survive in exceedingly low levels of Tti2 does suggest, however, that Tti2 may have a specialized role. This agrees with the limited number of Tti2 client proteins identified to date (only the PIKK proteins), the finding that Tti2 is not a heat shock protein, its relatively low cellular abundance (estimated to be up to 100-fold less than most of the heat shock proteins with the exception of Hsp42; Ghaemmaghami *et al.* 2003; refer to Table 3), and that overexpressing Hsp26, Hsp42, or Hsp104 fails to compensate for Tti2 depletion.

**Table 3:** Relative cellular abundance of TTT members to other heat shock proteins (Ghaemmaghami *et al.* 2003)

Protein	Molecules/Cell
Tel2	638
Tti1	721
Tti2	1470
Ssa1 (Hsp70)	269,000
Hsp82	445,000
Hsc82	132,000
Hsp104	32,800
Hsp26	19,300
Hsp42	1470

The nature of *tti2* alleles that suppress slow growth due to mutations in the FATC domain of Tra1 also suggests that the role for Tti2 in Tra1 biosynthesis is more complex. The C-terminal FATC domain of the PIKK proteins is integral to the kinase domain (Yang *et al.* 2013). Substitution of the terminal phenylalanine of Tra1 to alanine reduces both the level of the protein and its nuclear localization, particularly under stress. We described two alleles of *tti2* that almost completely reverse this effect (Genereaux *et al.* 2012). The suppressing alleles are partially dominant, but, since we have subsequently found numerous other suppressing alleles, many with multiple mutations, we believe that suppression is due to a loss of Tti2 function. If as predicted, partial loss of a Tti2 function restores the stability and localization of defective Tra1 derivatives, Tti2 would seem to have a role outside of protein folding. In this regard, the relationship between Tti2, Tel2, and Tti1 needs further investigation. Interestingly, our random selections for suppressors of *tra1_F3744A_* have identified numerous alleles of *tti2*, but not alleles of *tel2* or *tti1*.

The possibility that the PIKK proteins are the only known clients of the TTT complex has implications with regard to the function of the TTT complex. It also may further define the significance of the PIKK proteins, which may warrant their exclusive regulation by the TTT complex. What targets the PIKK proteins for regulation by Tti2 is unclear? Our earlier study highlighted a genetic interaction between the C-terminal FATC domain of Tra1 and Tti2 (Genereaux *et al.* 2012). The FATC domain is shared among the PIKK proteins, but is not likely to be solely responsible because suppression is not allele specific, and similar mutations in *mec1* are not suppressed (Genereaux *et al.* 2012). We also note that, to further address if there are additional clients of the TTT complex, we have analyzed two-dimensional gels before and after Tti2 depletion. Of the ∼500 proteins visible, we could detect none with a change in expression comparable to the Tra1 and Mec1, though two proteins, whose levels are reduced approximately three-fold upon Tti2 depletion, are being evaluated as potential clients.

## 

## Supplementary Material

Supplemental Material

## References

[bib1] Abraham R. T., 2004 PI 3-kinase related kinases: “Big” players in stress-induced signaling pathways. DNA Repair (Amst.) 3: 883–887. 15279773 10.1016/j.dnarep.2004.04.002

[bib2] Alberti S. Gitler A. D. Lindquist S., 2007 A suite of Gateway((R)) cloning vectors for high-throughput genetic analysis in *Saccharomyces cerevisiae*. Yeast 24: 913. 17583893 10.1002/yea.1502 PMC2190539

[bib3] Anderson C. M. Korkin D. Smith D. L. Makovets S. Seidel J. J., 2008 Tel2 mediates activation and localization of ATM/Tel1 kinase to a double-strand break. Genes Dev. 22: 854–859. 18334620 10.1101/gad.1646208 PMC2279195

[bib4] Brown C. M. Dalphin M. E. Stockwell P. A. Tate W. P., 1993 The translational termination signal database. Nucleic Acids Res. 21: 3119–3123. 8332534 10.1093/nar/21.13.3119 PMC309741

[bib5] Cashikar A. G. Duennwald M. Lindquist S. L., 2005 A chaperone pathway in protein disaggregation. Hsp26 alters the nature of protein aggregates to facilitate reactivation by Hsp104. J. Biol. Chem. 280: 23869–23875. 15845535 10.1074/jbc.M502854200 PMC1391974

[bib6] Cimprich K. A. Cortez D., 2008 ATR: an essential regulator of genome integrity. Nat. Rev. Mol. Cell Biol. 9: 616–627. 18594563 10.1038/nrm2450 PMC2663384

[bib7] Cross F. R., 1997 “Marker swap” plasmids: Convenient tools for budding yeast molecular genetics. Yeast 13: 647–653. 9200814 10.1002/(SICI)1097-0061(19970615)13:7<647::AID-YEA115>3.0.CO;2-#

[bib8] DaSilva L. F. Pillon S. Genereaux J. Davey M. J. Gloor G. B., 2013 The C-terminal residues of *Saccharomyces cerevisiae* Mec1 are required for its localization, stability, and function. G3 (Bethesda) 3: 1661–1674. 23934994 10.1534/g3.113.006841 PMC3789791

[bib9] Duennwald M. L. Lindquist S., 2008 Impaired ERAD and ER stress are early and specific events in polyglutamine toxicity. Genes Dev. 22: 3308–3319. 19015277 10.1101/gad.1673408 PMC2600758

[bib10] Duennwald M. L. Jagadish S. Muchowski P. J. Lindquist S., 2006 Flanking sequences profoundly alter polyglutamine toxicity in yeast. Proc. Natl. Acad. Sci. USA 103: 11045–11050. 16832050 10.1073/pnas.0604547103 PMC1544171

[bib11] Edelman I. Culbertson M. R., 1991 Exceptional codon recognition by the glutamine tRNAs in *Saccharomyces cerevisiae*. EMBO J. 10: 1481–1491. 2026145 10.1002/j.1460-2075.1991.tb07668.x PMC452811

[bib12] Genereaux J. Kvas S. Dobransky D. Karagiannis J. Gloor G. B., 2012 Genetic evidence links the ASTRA protein chaperone component tti2 to the SAGA transcription factor tra1. Genetics 191: 765–780. 22505622 10.1534/genetics.112.140459 PMC3389973

[bib13] Ghaemmaghami S. Huh W.-K. Bower K. Howson R. W. Belle A., 2003 Global analysis of protein expression in yeast. Nature 425: 737–741. 14562106 10.1038/nature02046

[bib15] Haslbeck M. Braun N. Stromer T. Richter B. Model N., 2004 Hsp42 is the general small heat shock protein in the cytosol of *Saccharomyces cerevisiae*. EMBO J. 23: 638–649. 14749732 10.1038/sj.emboj.7600080 PMC1271810

[bib16] Haslbeck M. Walke S. Stromer T. Ehrnsperger M. White H. E., 1999 Hsp26: a temperature-regulated chaperone. EMBO J. 18: 6744–6751. 10581247 10.1093/emboj/18.23.6744 PMC1171736

[bib17] Hoffman C. S. Winston F., 1987 A ten-minute DNA preparation from yeast efficiently releases autonomous plasmids for transformation of *Escherichia coli*. Gene 57: 267–272. 3319781 10.1016/0378-1119(87)90131-4

[bib18] Hoke S. M. T. Irina Mutiu A. Genereaux J. Kvas S. Buck M., 2010 Mutational analysis of the C-terminal FATC domain of *Saccharomyces cerevisiae* Tra1. Curr. Genet. 56: 447–465. 20635087 10.1007/s00294-010-0313-3 PMC2943577

[bib19] Hořejší Z. Takai H. Adelman C. A. Collis S. J. Flynn H., 2010 CK2 phospho-dependent binding of R2TP complex to TEL2 is essential for mTOR and SMG1 stability. Mol. Cell 39: 839–850. 20864032 10.1016/j.molcel.2010.08.037

[bib20] Huisman O. Raymond W. Froehlich K. U. Errada P. Kleckner N., 1987 A Tn10-lacZ-kanR-URA3 gene fusion transposon for insertion mutagenesis and fusion analysis of yeast and bacterial genes. Genetics 116: 191–199. 3038670 10.1093/genetics/116.2.191 PMC1203129

[bib21] Hurov K. E. Cotta-Ramusino C. Elledge S. J., 2010 A genetic screen identifies the Triple T complex required for DNA damage signaling and ATM and ATR stability. Genes Dev. 24: 1939–1950. 20810650 10.1101/gad.1934210 PMC2932975

[bib23] Kopczynski J. B. Raff A. C. Bonner J. J., 1992 Translational readthrough at nonsense mutations in the HSF1 gene of *Saccharomyces cerevisiae*. MGG Mol. Gen. Genet. 234: 369–378. 1406583 10.1007/BF00538696

[bib24] Kryndushkin D. S. Alexandrov I. M. Ter-Avanesyan M. D. Kushnirov V. V., 2003 Yeast [PSI+] prion aggregates are formed by small Sup35 polymers fragmented by Hsp104. J. Biol. Chem. 278: 49636–49643. 14507919 10.1074/jbc.M307996200

[bib25] Langouët M. Saadi A. Rieunier G. Moutton S. Siquier-Pernet K., 2013 Mutation in TTI2 reveals a role for triple T complex in human brain development. Hum. Mutat. 34: 1472–1476. 23956177 10.1002/humu.22399

[bib26] Lempiäinen H. Halazonetis T. D., 2009 Emerging common themes in regulation of PIKKs and PI3Ks. EMBO J. 28: 3067–3073. 19779456 10.1038/emboj.2009.281 PMC2752028

[bib27] Lovejoy C. A. Cortez D., 2009 Common mechanisms of PIKK regulation. DNA Repair (Amst.) 8: 1004–1008. 19464237 10.1016/j.dnarep.2009.04.006 PMC2725225

[bib28] Lustig A. J. Petes T. D., 1986 Identification of yeast mutants with altered telomere structure. Proc. Natl. Acad. Sci. USA 83: 1398–1402. 3513174 10.1073/pnas.83.5.1398 PMC323083

[bib29] Mutiu A. I. Hoke S. M. T. Genereaux J. Hannam C. MacKenzie K., 2007 Structure/function analysis of the phosphatidylinositol-3-kinase domain of yeast Tra1. Genetics 177: 151–166. 17660562 10.1534/genetics.107.074476 PMC2013730

[bib30] Najmabadi H. Hu H. Garshasbi M. Zemojtel T. Abedini S. S., 2011 Deep sequencing reveals 50 novel genes for recessive cognitive disorders. Nature 478: 57–63. 21937992 10.1038/nature10423

[bib47] Pal M. Morgan M. Phelps S. E. L. Roe S. M. Parry-Morris S., 2014 Structural basis for phosphorylation-dependent recruitment of Tel2 to Hsp90 by Pih1. Structure 22: 805–818. 24794838 10.1016/j.str.2014.04.001 PMC4058522

[bib31] Parsell D. A. Kowal A. S. Singer M. A. Lindquist S., 1994 Protein disaggregation mediated by heat-shock protein Hsp104. Nature 372: 475–478. 7984243 10.1038/372475a0

[bib32] Pure G. A. Robinson G. W. Naumovski L. Friedberg E. C., 1985 Partial suppression of an ochre mutation in *Saccharomyces cerevisiae* by multicopy plasmids containing a normal yeast tRNAGln gene. J. Mol. Biol. 183: 31–42. 2989539 10.1016/0022-2836(85)90278-5

[bib33] Runge K. W. Zakian V. A., 1996 TEL2, an essential gene required for telomere length regulation and telomere position effect in *Saccharomyces cerevisiae*. Mol. Cell. Biol. 16: 3094–3105. 8649421 10.1128/mcb.16.6.3094 PMC231304

[bib34] Shevchenko A. Roguev A. Schaft D. Buchanan L. Habermann B., 2008 Chromatin Central: towards the comparative proteome by accurate mapping of the yeast proteomic environment. Genome Biol. 9: R167. 19040720 10.1186/gb-2008-9-11-r167 PMC2614481

[bib35] Shimobayashi M. Hall M. N., 2014 Making new contacts: the mTOR network in metabolism and signalling crosstalk. Nat. Rev. Mol. Cell Biol. 15: 155–162. 24556838 10.1038/nrm3757

[bib36] Stirling P. C. Bloom M. S. Solanki-Patil T. Smith S. Sipahimalani P., 2011 The complete spectrum of yeast chromosome instability genes identifies candidate cin cancer genes and functional roles for astra complex components. PLoS Genet. 7: e1002057. 21552543 10.1371/journal.pgen.1002057 PMC3084213

[bib37] Stromer T. Ehrnsperger M. Gaestel M. Buchner J., 2003 Analysis of the interaction of small heat shock proteins with unfolding proteins. J. Biol. Chem. 278: 18015–18021. 12637495 10.1074/jbc.M301640200

[bib38] Takai H. Wang R. C. Takai K. K. Yang H. de Lange T., 2007 Tel2 regulates the stability of PI3K-related protein kinases. Cell 131: 1248–1259. 18160036 10.1016/j.cell.2007.10.052

[bib39] Takai H. Xie Y. De Lange T. Pavletich N. P., 2010 Tel2 structure and function in the Hsp90-dependent maturation of mTOR and ATR complexes. Genes Dev. 24: 2019–2030. 20801936 10.1101/gad.1956410 PMC2939364

[bib41] von der Haar T., 2007 Optimized protein extraction for quantitative proteomics of yeasts. PLoS One 2: e1078. 17957260 10.1371/journal.pone.0001078 PMC2031916

[bib42] von der Haar T. Tuite M. F., 2007 Regulated translational bypass of stop codons in yeast. Trends Microbiol. 15: 78–86. 17187982 10.1016/j.tim.2006.12.002

[bib43] Weinert T. A. Kiser G. L. Hartwell L. H., 1994 Mitotic checkpoint genes in budding yeast and the dependence of mitosis on DNA replication and repair. Genes Dev. 8: 652–665. 7926756 10.1101/gad.8.6.652

[bib44] Winzeler E. A. Davis R. W., 1997 Functional analysis of the yeast genome. Curr. Opin. Genet. Dev. 7: 771–776. 9468786 10.1016/s0959-437x(97)80039-1

[bib45] Yamashita A. Kashima I. Ohno S., 2005 The role of SMG-1 in nonsense-mediated mRNA decay. Biochim. Biophys. Acta. Proteins Proteomics 1754: 305–315. 10.1016/j.bbapap.2005.10.002 16289965

[bib46] Yang H. Rudge D. G. Koos J. D. Vaidialingam B. Yang H. J., 2013 mTOR kinase structure, mechanism and regulation. Nature 497: 217–223. 23636326 10.1038/nature12122 PMC4512754

