# Supplementary material for: Saccharomyces cerevisiae Tti2 Regulates PIKK Proteins and Stress Response
Source: G3 (Bethesda). 2016 Apr 5;6(6):1649–59. doi: 10.1534/g3.116.029520 (PMC4889661; doi:10.1534/g3.116.029520)
Supplement: Supplemental Material [file supp_6_6_1649__index.html]

Saccharomyces cerevisiae Tti2 Regulates PIKK Proteins and Stress Response — Saccharomyces cerevisiae Tti2 Regulates PIKK Proteins and Stress Response — Supplemental Material 

# *Saccharomyces cerevisiae* Tti2 Regulates PIKK Proteins and Stress Response

## Supplemental Material for Hoffmann *et al.*, 2016

**Files in this Data Supplement:**

- Figure S1 - Selection of randomly created *tti2* mutations that cause slow growth. (.pdf, 124 KB)
- Figure S2 - *GAL10-TTI2* expression in galactose and raffinose containing media. (.pdf, 100 KB)
- Figure S3 - Relative levels of constitutive and depleted *Tti2* expression. (.pdf, 104 KB)
- Figure S4 - Depleting Tti2 does not result in aggregation of Mec1. (.pdf, 111 KB)
- Figure S5 - Synthetic slow growth due to exon 1 of the human Huntingtin gene containing a 103 residue polyQ sequence. (.pdf, 90 KB)
- Figure S6 - Overexpression of HSP26, HSP104 or HSPHSP42 does not compensate for depleting Tti2. (.pdf, 169 KB)
- Table S1 - Strains used in this study. (.pdf, 263 KB)
- Table S2 - Oligonucleotides used in this study. (.pdf, 117 KB)
