# Supplementary material for: Saccharomyces cerevisiae Tti2 Regulates PIKK Proteins and Stress Response
Source: G3 (Bethesda). 2016 Apr 5;6(6):1649–59. doi: 10.1534/g3.116.029520 (PMC4889661; doi:10.1534/g3.116.029520)
Supplement: Supplemental Material [file supp_g3.116.029520_FigureS1.pdf]

Sequence of oligonucleotide to integrate C-terminal TTI2 Myc^9-tag.

XbaI SphI
GCTCTAGAGCATGCGACATTTTATCATTAATTCTAGTTACATACGATAAGTGTTCGCAAGAGGATGCGTTGAACGAAT
CAATACTACAACAATGTAAGGAAACGATAAGCTGGTTGCTGAATTGTGACTGCGCTATGGGTGAACAGTTATCGACT
CTATCTAAGCAACCGCGGTTTC
SalI HindIII NotI
AGTTACTTTTTGAGTTCTCAGTCGACAAGCTTGGAACCGCGGCCGCCTTCTCATAGTCTAGGATAGAGACACAGAC
ACAGACAAATGCATATACATATATATATATATATAACTAGAC
BamHI
AGTATATAAACAAAAAGAGTTATTTGGATCCCTTTATGCTGAAGAATCCCTTTTAAAGAAACCTTGGACATATATTAAAA
TATTCATTTGCTTATTTACAGATATATTAAGGTAAAGAGAATAATGACTCGGAGAGATAAATCGGTTTCATATTAACGGG
TGTTTTGCAACCCGTTCACAATCCTGATATTAGTATCCAAAAACCGATTCACACAGTTAGACAAACATTGCTCTTC
SacI EcoRI
TTGCGAGCTCGAATTCAC

Figure S1. *Sequence of the DNA used to integrate a C-terminal Myc^9-tag into TTI2.*
