# Supplementary material for: Saccharomyces cerevisiae Tti2 Regulates PIKK Proteins and Stress Response
Source: G3 (Bethesda). 2016 Apr 5;6(6):1649–59. doi: 10.1534/g3.116.029520 (PMC4889661; doi:10.1534/g3.116.029520)
Supplement: Supplemental Material [file supp_g3.116.029520_FigureS2.pdf]

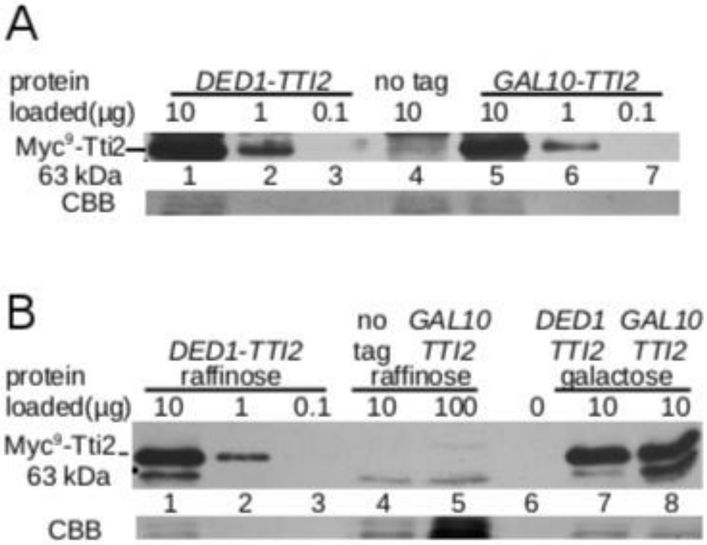

Figure S2. *GAL10-TTI2 expression in galactose and raffinose containing media*. **A.** *GAL10-TTI2* expression in galactose-containing medium. Yeast strain CY6070 (lanes 1, 2, and 3), CY6971 (lanes 5, 6, and 7), and BY4742 (no tag control; lane 4) were grown to stationary phase in galactose-containing YP, diluted 1:20 in fresh medium and grown for 8 hours before harvesting. Protein was extracted with glass beads and the indicated amounts separated by SDS-PAGE and Western blotted with anti-Myc antibody to detect tagged Tti2. Lane numbers are listed between the blot and the bottom of the gel, which was stained with Coomassie Brilliant Blue for a loading control (CBB). **B.** *GAL10-TTI2* in raffinose medium. Strains CY6070, CY6971 and BY4742 were prepared for Western blotting as in (A), with the exception that galactose was substituted by raffinose.
