# Supplementary material for: Saccharomyces cerevisiae Tti2 Regulates PIKK Proteins and Stress Response
Source: G3 (Bethesda). 2016 Apr 5;6(6):1649–59. doi: 10.1534/g3.116.029520 (PMC4889661; doi:10.1534/g3.116.029520)
Supplement: Supplemental Material [file supp_g3.116.029520_FigureS3.pdf]

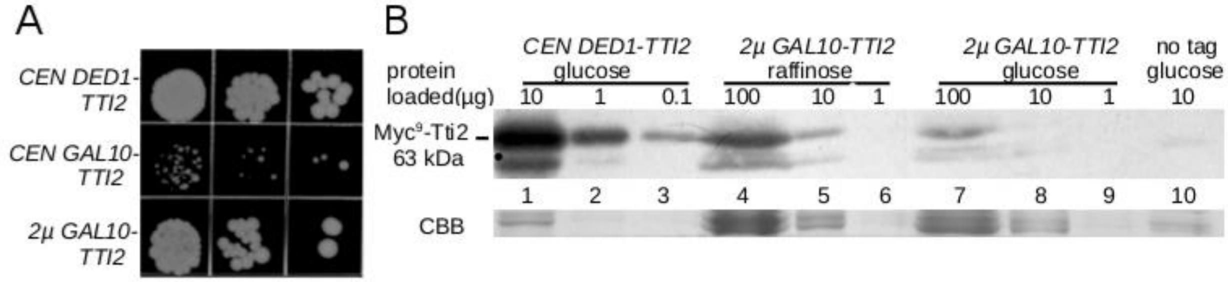

Figure S3. *Relative levels of constitutive and depleted Tti2 expression.* **A.** CY6070, CY6971 (*CEN GAL10-TTI2*), and CY6991 (*2µ GAL10-TTI2*) were grown to stationary phase in YPD medium, cell densities were normalized, then spotted in 10-fold serial dilutions onto a YPD plate and grown for three days at 30º. **B.** Yeast strains CY6070 (lanes 1, 2, and 3), CY6991 (lanes 4-9), and BY4742 (no tag control; lane 10) were grown to stationary phase in YP media containing the indicated carbon source, diluted 1:20 in the same media, then grown for 8 hours. Cells were lysed using glass beads and protein extracts separated by SDS-PAGE. Western blotting was then performed using an anti-Myc antibody. The bottom of the gel was stained with Coomassie Brilliant Blue and shown as a loading control.
