# Supplementary material for: Saccharomyces cerevisiae Tti2 Regulates PIKK Proteins and Stress Response
Source: G3 (Bethesda). 2016 Apr 5;6(6):1649–59. doi: 10.1534/g3.116.029520 (PMC4889661; doi:10.1534/g3.116.029520)
Supplement: Supplemental Material [file supp_g3.116.029520_FigureS4.pdf]

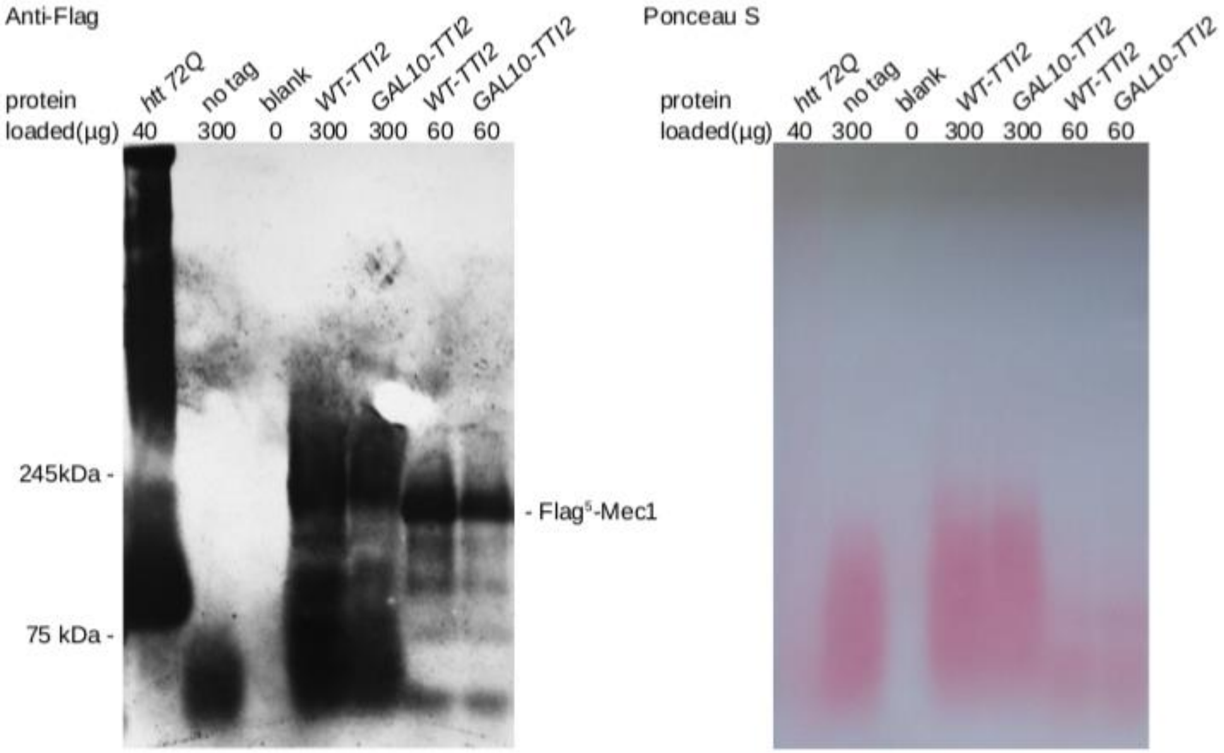

Figure S4. *Depleting Tti2 does not result in aggregation of Mec1*. Yeast strain CY6194, CY7030, and BY4742 were grown in raffinose containing medium then diluted 1:20 in YPD and grown for 8 hours. Protein was extracted by bead lysis and the indicated amounts separated on a 1.8% agarose gel containing 0.1% SDS. Protein was transferred onto a PVDF membrane by capillary transfer and Western blotted using an anti-Flag antibody (left). A W303 yeast strain expressing human huntinton exon I variant containing a 72 residue polyQ region was used as a positive control (described in Duennwald *et al.* 2006). The membrane was stained with Ponceau S prior to Western blotting and shown for a loading control (right).
