# Supplementary material for: Saccharomyces cerevisiae Tti2 Regulates PIKK Proteins and Stress Response
Source: G3 (Bethesda). 2016 Apr 5;6(6):1649–59. doi: 10.1534/g3.116.029520 (PMC4889661; doi:10.1534/g3.116.029520)
Supplement: Supplemental Material [file supp_g3.116.029520_FigureS5.pdf]

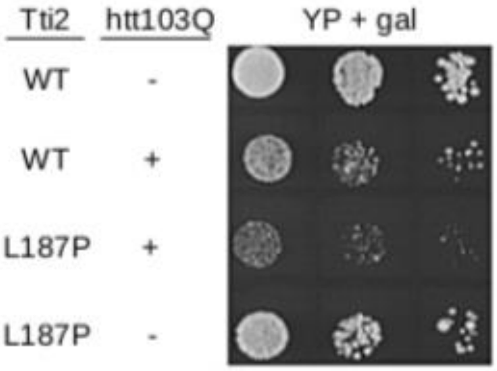

Figure S5. *Synthetic slow growth due to exon 1 of the human Huntingtin gene containing a 103 residue polyQ sequence.* The wild-type (CY6857) and *tti2*$_{L187P}$ (CY6872) strains were transformed with YCplac33 or *CEN-GAL1-htt103Q*, and grown to stationary phase in minimal medium containing 2% glucose and lacking uracil. Cell densities were normalized then cells spotted in 10-fold serial dilutions onto a YP plate containing 2% galactose and grown at 30° for two days.
