# Supplementary material for: Saccharomyces cerevisiae Tti2 Regulates PIKK Proteins and Stress Response
Source: G3 (Bethesda). 2016 Apr 5;6(6):1649–59. doi: 10.1534/g3.116.029520 (PMC4889661; doi:10.1534/g3.116.029520)
Supplement: Supplemental Material [file supp_g3.116.029520_FigureS6.pdf]

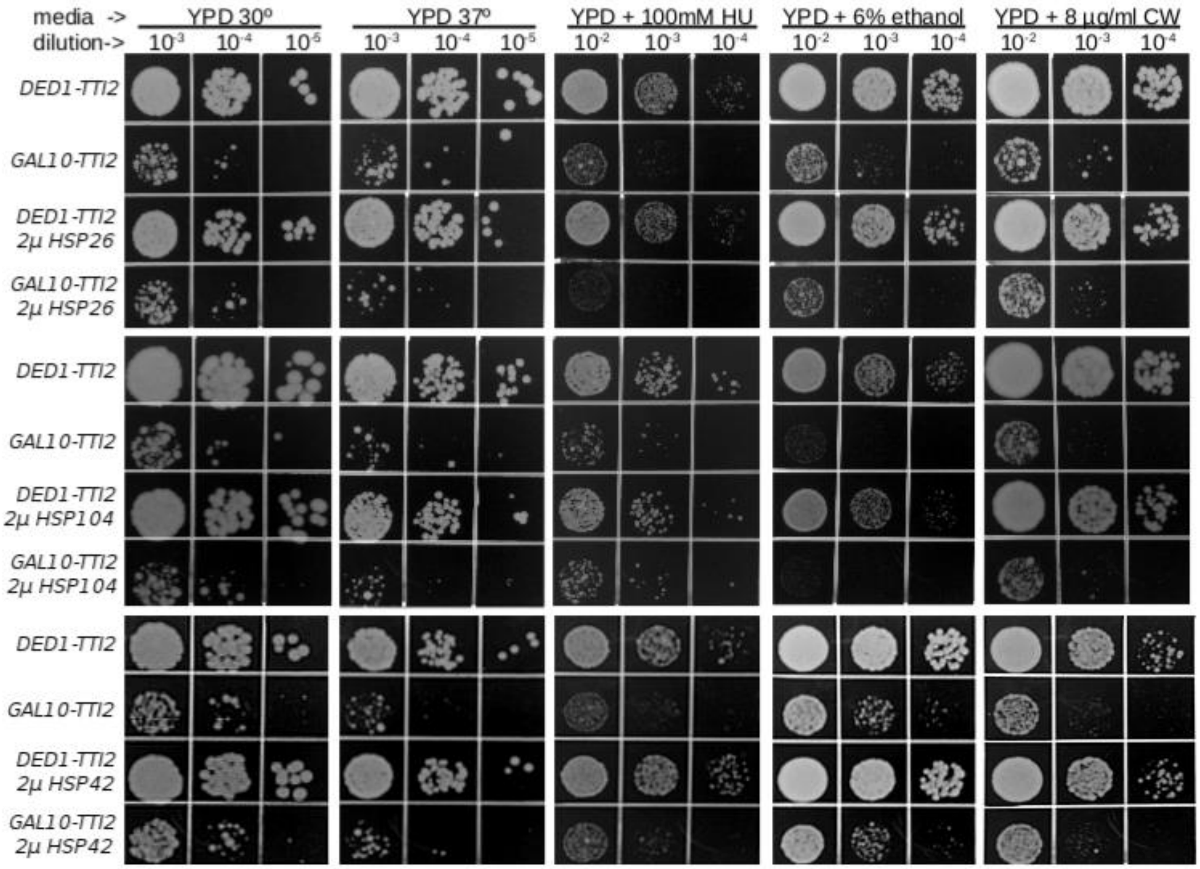

Figure S6. *Overexpression of HSP26, HSP104 or HSPHSP42 does not compensate for depleting Tti2*. Strains CY7245 (*DED1-TTI2* YCplac33), CY7249 (*GAL10-TTI2* YCp33), CY7247 (*DED1-TTI2 2µ-GPD-HSP26*), CY7251 (*GAL10-TTI2 2µ-GPD-HSP26*), CY7248 (*DED1-TTI2 2µ-GPD-HSP104*), CY7252 (*GAL10-TTI2 2µ-GPD-HSP104*), CY7323 (*DED1-TTI2 2µ-HSP42-HSP42*), and CY7324 (*GAL10-TTI2 2µ-HSP42-HSP42*) were grown to stationary phase in medium lacking uracil and containing raffinose. Cell densities were normalized and then 10-fold serial dilutions spotted onto YPD plates grown at either 30º or 37º, and onto YPD plates containing 6% ethanol, 8 µg/ml Calcofluor white, or 100 mM hydroxyurea.
