# Supplementary material for: Saccharomyces cerevisiae Tti2 Regulates PIKK Proteins and Stress Response
Source: G3 (Bethesda). 2016 Apr 5;6(6):1649–59. doi: 10.1534/g3.116.029520 (PMC4889661; doi:10.1534/g3.116.029520)
Supplement: Supplemental Material [file supp_g3.116.029520_TableS1.pdf]

Table S1. Strains used in this study.

Strain	Genotype	Plasmid (plasmid-promoter-gene)	Reference
BY4741	*MATa his3Δ0 leu2Δ0 Δmet15 ura3Δ0*		(Winzeler and Davis 1997)
BY4742	*MATα his3Δ0 leu2Δ0 lys2Δ0 ura3Δ0*		(Winzeler and Davis 1997)
BY4743	*MATa/α his3Δ1/his3Δ1 leu2Δ0/leu2Δ0 LYS2/lys2Δ0 met15Δ0/MET15 ura3Δ0/ura3Δ0*		(Winzeler and Davis 1997)
CY1217	*MATa his3Δ0 leu2Δ0 Δmet15 ura3Δ0 Δire1::kanMX*		(Tong *et al*. 2001)
CY5857	*mec2-1*		(Weinert *et al*, 1994)
CY5919	*MATa his3Δ0 leu2Δ0 ura3Δ0 URA3-Flag*^{5}*-TRA1*		(Genereaux *et al.* 2012)
CY5998	Isogenic to BY4742 except *URA3-eGFP-TRA1*		(Genereaux *et al.* 2012)
CY6032	Isogenic to BY4743 except *tti2Δ-met5Δ::*Tn*10*luk		This work
CY6049	*MATa his3Δ0 leu2Δ0 met5Δ0 ura3Δ0 tti2Δ-met5Δ::*Tn*10*luk	YCplac111-*DED1-TTI2*	This work
CY6070	*MATa his3Δ0 leu2Δ0 met5Δ0 ura3Δ0 tti2Δ-met5Δ::*Tn*10*luk	YCplac33-*DED1-TTI2*	This work
CY6194	*MATa his3Δ0 leu2Δ0 ura3Δ0 URA3-Flag*^{5}*-MEC1*		(DaSilva *et al.* 2013)
CY6306	*MATa ura3Δ0 his3Δ0 leu2Δ0 URA3-eGFP-MEC1*		(DaSilva *et al.* 2013)
CY6415	Isogenic to BY4743 except *TOR1/URA3-Flag*^{5}*-TOR1*		This work
CY6767	Spore colony of CY4162 *MATa his3Δ0 leu2Δ0 tel2Δ::KanMX*	YCplac33-*DED1-TEL2*	This work
CY6857	Isogenic to CY6070	YCplac111-*DED1-TTI2*	This work
CY6872	Isogenic to CY6070	YCplac111-*DED1-tti2*$_{L187P}$	This work
CY6874	Isogenic to CY6070	YCplac111-*DED1-tti2*$_{Q276TAA}$	This work
CY6963	*MATα his3Δ0 leu2Δ0 ura3Δ0 tti2Δ-met5Δ::*Tn*10*luk	YCplac33-*DED1-TTI2*	This work
CY6971	Isogenic to CY6070	YCplac111-*GAL10-TTI2*	This work

CY6973	Isogenic to CY6070	YCplac111-*GAL10-TTI2-leu2Δ0::URA3*	This work
CY6991	Isogenic to CY6070	YEplac181-*GAL10-TTI2*	This work
CY6999	Isogenic to CY6971 except *URA3-Flag*^{5}*-TRA1*	YCplac111-*GAL10-TTI2*	This work
CY7000	Diploid of CY6971 and CY6963	YCplac111-*GAL10-TTI2*	This work
CY7030	Isogenic to CY6971 except *URA3-Flag*^{5}*-MEC1*	YCplac111-*GAL10-TTI2*	This work
CY7035	Diploid of CY6963 x CY6965 with *TOR1*/*URA3-Flag*^{5}*-TOR1*	YCplac111-*GAL10-TTI2*	This work
CY7086	Isogenic to BY4742 except *TTI2-Myc*^{9}*-HIS3*		This work
CY7172	Isogenic to CY6971	YCplac33-*TTI2-TTI2*	This work
CY7189	Isogenic to CY6971 except *URA3-eGFP-MEC1*	YCplac111-*GAL10-TTI2*	This work
CY7193	Isogenic to CY6971 except *URA3-eGFP-TRA1*	YCplac111-*GAL10-TTI2*	This work
CY7236	Isogenic to CY6857	*2μ URA3 GAL1-FLAG-htt25Q*	This work
CY7237	Isogenic to CY6857	*2μ URA3 GAL1-FLAG-htt25Q*	This work
CY7238	Isogenic to CY6872	*2μ URA3 GAL1-FLAG-htt103Q*	This work
CY7239	Isogenic to CY6872	*2μ URA3 GAL1-FLAG-htt103Q*	This work
CY7241	Isogenic to CY6872	YCplac33	This work
CY7245	Isogenic to CY6857	YCplac33	This work
CY7247	Isogenic to CY6857	*2μ URA3 GPD-HSP26*	This work
CY7248	Isogenic to CY6857	*2μ URA3 GPD-HSP104*	This work
CY7249	Isogenic to CY6971	YCplac33	This work
CY7251	Isogenic to CY6971	*2μ URA3 GPD-HSP26*	This work
CY7252	Isogenic to CY6971	*2μ URA3 GPD-HSP104*	This work
CY7323	Isogenic to CY6857	*2μ URA3 HSP42-HSP42*	This work
CY7324	Isogenic to CY6971	*2μ URA3 HSP42-HSP42*	This work
CY7370	Isogenic to CY6070	*2μ LEU2 GPD-CDC37*	This work
CY7371	Isogenic to CY6070	*2μ LEU2 GPD-HSP82*	This work
CY7372	Isogenic to CY6070	*2μ LEU2 GPD-AHA1*	This work
CY7373	Isogenic to CY6070	*2μ LEU2 GPD-HSC82*	This work
CY7374	Isogenic to CY6973	*2μ LEU2 GPD-CDC37*	This work
CY7375	Isogenic to CY6973	*2μ LEU2 GPD-HSP82*	This work
CY7376	Isogenic to CY6973	*2μ LEU2 GPD-AHA1*	This work
CY7377	Isogenic to CY6973	*2μ LEU2 GPD-HSC82*	This work
