# Supplementary material for: Saccharomyces cerevisiae Tti2 Regulates PIKK Proteins and Stress Response
Source: G3 (Bethesda). 2016 Apr 5;6(6):1649–59. doi: 10.1534/g3.116.029520 (PMC4889661; doi:10.1534/g3.116.029520)
Supplement: Supplemental Material [file supp_g3.116.029520_TableS2.pdf]

Table S2. Oligonucleotides used in this study.

Name	Sequence (5'-3')	Description
2764-1	AACTGCAGTAATACGCTTAACTGCTC	*GAL10* promoter
2764-2	CCCAAGCTTGACGTTAAAGTATAGAGGT	*GAL10* promoter
4213-1	GGCCGACTACAAGGACGACGATGACAAGGC	Flag tag
4213-2	GGCCGCCTTGTCATCGTCGTCCTTGTAGTC	Flag tag
5693-1	ATAAGAATGCGGCCGCAATGACGGCCGTAACTGATATC	*TTI2*
5693-2	ATACGAGCTCTGCATTTGTCTGTGTCTGTGT	*TTI2*
6085-1	CCTGTCACCATATGACGCGG	*TTI2*
6085-2	CAGGATCCACTACCTGCTCGATGTTCC	*TTI2*
6085-3	GGGGATCCGGAAACGATAAGCTGGTTGC	*TTI2*
6085-4	GGTTGGTCGACGGGATTCTTCAGCATAAAGGG	*TTI2*
6496-1	ACATGCATGCTACTAAAGCCTCATTTCCAA	*TOR1*
6496-2	CCCAAGCTTAAACAATCCCGCTGTTGT	*TOR1*
6496-3	ATAAGAATGCGGCCGCTATGGAACCGCATGAGGAGC	*TOR1*
6496-4	ACGCGTCGACAATTCATGTTCCAAGGA	*TOR1*
TD0569	GCCAAGCTTATATTTTGTTATATGATTTTATTCTTCACC	*TTI2* promoter
TD0570	TGGTAAAGCTTCACATACTTATAACTTGATGC	*TTI2* promoter
MD11	GGGGACAAGTTTGTACAAAAAAGCAGGCTATGGCCATTGATTACTCTAAGTGG	*CDC37*
MD12	GGGGACCACTTTGTACAAGAAAGCTGGGTCTAGTCAACAGTGTCGGCAGTATG	*CDC37*
MD13	GGGGACAAGTTTGTACAAAAAAGCAGGCTATGGCTAGTGAAACTTTTGAATTTC	*HSP82*
MD14	GGGGACCACTTTGTACAAGAAAGCTGGGTCTAATCTACCTCTTCCATTTCGGT	*HSP82*
MD15	GGGGACAAGTTTGTACAAAAAAGCAGGCTATGGTCGTGAATAACCCAAATAAC	*AHA1*
MD16	GGGGACCACTTTGTACAAGAAAGCTGGGTTTATAATACGGCACCAAAGCC	*AHA1*
MD17	GGGGACAAGTTTGTACAAAAAAGCAGGCTATGGCTGGTGAAACTTTTGAA	*HSC82*

MD18	GGGGACCACTTTGTACAAGAAAGCTGGGTTTAATCAACTTCTTCCATCTCGG	*HSC82*
TK7578	AAGGAAAAAAGCGGCCGCTTCCAAATTTTTAATATTTAGCTGGG	*HSP42*
TK7589	CCCGAGCTCTCGTTGTATGATTTTTGTGTGGT	*HSP42*
